# Color‐Tunable Room‐Temperature Phosphorescence from Non‐Aromatic‐Polymer‐Involved Charge Transfer

**DOI:** 10.1002/advs.202404698

**Published:** 2024-06-14

**Authors:** Ningyan Li, Xipeng Yang, Binbin Wang, Panyi Chen, Yixian Ma, Qianqian Zhang, Yiyao Huang, Yan Zhang, Shaoyu Lü

**Affiliations:** ^1^ State Key Laboratory of Applied Organic Chemistry Lanzhou Magnetic Resonance Center Department of Chemistry and Chemical Engineering Lanzhou University Lanzhou 730000 China

**Keywords:** charge transfer, color‐tunable, non‐aromatic polymer, quinoline zwitterion, room‐temperature phosphorescence

## Abstract

Polymeric room‐temperature phosphorescence (RTP) materials especially multicolor RTP systems hold great promise in concrete applications. A key feature in these applications is a triplet charge transfer transition. Aromatic electron donors and electron acceptors are often essential to ensure persistent RTP. There is much interest in fabricating non‐aromatic charge‐transfer‐mediated RTP materials and it still remains a formidable challenge to achieve color‐tunable RTP via charge transfer. Herein, a charge‐transfer‐mediated RTP material by embedding quinoline derivatives within a non‐aromatic polymer matrix such as polyacrylamide (PAM) or polyvinyl alcohol (PVA) is developed. Through‐space charge transfer (TSCT) is achieved upon alkali‐ or heat treatment to realize a long phosphorescence lifetime of up to 629.90 ms, high phosphorescence quantum yield of up to 20.51%, and a green‐to‐blue afterglow for more than 20 s at room temperature. This color‐tunable RTP emerges from a nonaromatic polymer to single phosphor charge transfer that has rarely been reported before. This finding suggests that an effective and simple approach can deliver new color‐tunable RTP materials for applications including multicolor display, information encryption, and gas detection.

## Graphical abstract

A color‐tunable room‐temperature phosphorescence (RTP) material is achieved by embedding quinoline zwitterion within a non‐aromatic polyacrylamide (PAM) matrix upon alkali‐ or heat treatment. Electron‐deficient chromophores (acceptor, A) combine with the electron‐rich hydroxide or amide groups (donor, D) via through‐space charge transfer (TSCT) to form a new emission center and to achieve the blue shift of phosphorescence and long‐lived RTP.

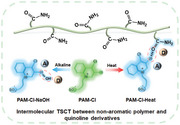

## 1 Introduction

Room‐temperature phosphorescence (RTP) materials hold a prominent position in the fast‐growing domain of luminescent materials,^[^ ^1^ ^]^ owing to their great potential in the fields of information storage,^[^ ^2^ ^]^ detection,^[^ ^2^, ^3^ ^]^ anti‐counterfeiting,^[^ ^4^ ^]^ bioimaging^[^ ^5^ ^]^ and optoelectronic devices.^[^ ^6^ ^]^ Their unique triplet emission endows an extraordinary afterglow visible to the naked eye at room temperature.^[^ ^7^ ^]^ However, a single phosphorescent emission color is usually observed, so that the material requirements for some demanding applications have not yet been met. Achieving color‐tunable RTP materials has therefore been a major goal in recent studies.^[^ ^4^, ^8^ ^]^

Polymeric RTP materials are an intriguing sub‐class of RTP materials since they have the advantages of excellent bio‐compatibility, high flexibility, structural diversity, and relatively low cost, compared to inorganic counterparts.^[^ ^9^ ^]^ The polymeric RTP systems have been achieved by doping small organic phosphors into polymers to improve the photophysical properties.^[^ ^10^ ^]^ The RTP phenomenon is known to be due to the restrained molecular motions of excited‐state phosphors by a rigid polymer matrix.^[^ ^11^ ^]^ In the meantime, polymers are rich in hydrogen bonds or reactive functional groups that can interact with the doping molecules to obtain color‐tunable RTP,^[^ ^12^ ^]^ where strategies include building dynamic covalent bonds,^[^ ^13^ ^]^ forming multiple aggregates,^[^ ^14^ ^]^ fabricating multiple phosphorescent emitters,^[^ ^15^ ^]^ and replacing diverse phosphorescent monomers.^[^ ^6^ ^]^ Although promising strides have been made, achieving color‐tunable RTP of a single phosphor in polymeric materials using an effective and simple approach remains a relevant challenge.

**Figure 1:**
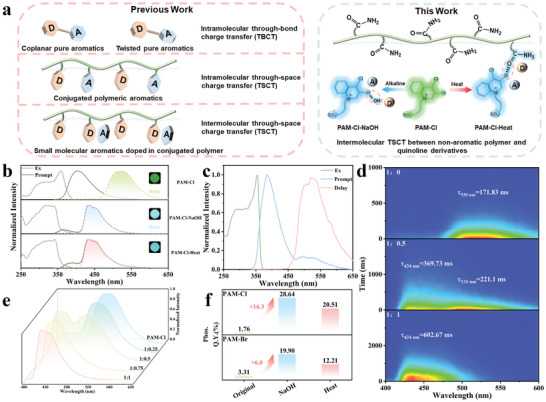
a) Design sketch of the charge‐transfer‐mediated RTP systems in previous works and this work. b) Excitation spectra (green solid line), prompt (brown solid line), and delayed (dash line) phosphorescence spectra of PAM‐Cl, PAM‐Cl‐NaOH, and PAM‐Cl‐Heat. Delayed time: 1.0 ms. c) Excitation spectra, prompt and delayed phosphorescence spectra of 4‐Cl (1✗10^‐6^ M) in methanol at 77 K. d) The time‐resolved emission spectra (excited at 340 nm) of PAM‐Cl‐NaOH treated with 0, 0.5, and 1 equivalent of NaOH and their phosphorescence lifetime. e) Normalized phosphorescence emission spectra of PAM‐Cl‐NaOH with 0, 0.25, 0.50, 0.75, and 1 equivalent of NaOH. f) The changes in phosphorescence quantum yield of PAM‐Cl and PAM‐Br treated with NaOH or heat.

In the last decade, charge transfer (CT) interactions, formed between a donor (D) and an acceptor (A), have been harnessed to form triplet excitons for light emission.^[^ ^16^ ^]^ The strong interactions can effectively suppress nonradiative decay, leading to significantly enhanced photoluminescence quantum yields.^[^ ^17^ ^]^ However, one of the most critical parameters for electronic coupling is the suitable distance between the D and A moieties to allow for efficient direct absorption by the CT state.^[^ ^16b^ ^]^ Therefore, conjugated D‐A architectures have been widely developed via through‐bond charge transfer (TBCT) (**Figure** **1**a). In 2015, CT aromatics composed of a carbazole donor and a triazine acceptor were reported by Huang et al..^[^ ^18^ ^]^ Ultralong‐lived luminescence (1.06 s) with a quantum efficiency of 1.25% at room temperature was achieved. By tailoring the structure of D and A, the color of RTP can be tuned from blue‐green to red. Recently, through‐space charge transfer (TSCT) has been employed to access long‐lived ^3^CT state and high triplet energy (Figure 1a), such as for organic small molecules comprising 9,9‐dimethylacridine and naphthalimide as the D and A units, respectively.^[^ ^19^ ^]^ The single‐molecular RTP material exhibited a long lifetime of 210 ms and a high ^3^CT energy level of 2.50 eV due to relatively weaker electron coupling between D and A species. Zhang et al. reported charge‐transfer‐mediated RTP by doping a poly(N,N‐dimethylaninline) (PDMA) donor with a pyrene derivatives acceptor.^[^ ^20^ ^]^ The RTP showed red afterglow with a duration of 5 s. In comparison to these aromatic systems (both D and A units are aromatics), non‐aromatic charge‐transfer‐mediated RTP has been rarely achieved before.^[^ ^21^ ^]^

Here we introduce a charge‐transfer‐mediated RTP material by embedding quinoline derivatives within non‐aromatic polymer matrix such as polyacrylamide (PAM) or polyvinyl alcohol (PVA). The rigid polymer matrix promotes the intersystem crossing ISC process efficiently and inhibits the non‐radiative transition of the triplet excitons.^[^ ^22^ ^]^ With alkali‐ or heat treatment, electron‐deficient chromophores combine with the electron‐rich groups (hydroxide, amide, or hydroxy groups) to form a new emission center via charge transfer and to achieve the blue shift of phosphorescence (Figure 1a). In addition, the formation of charge transfer interactions further limits the movement of the triplet excitons and improves the phosphorescent performance, thus producing long‐lived RTP up to 629.90 ms, quantum yield (Φ_P_) up to 20.51%, and the blue afterglow for more than 20 s at room temperature. In the following, a detailed mechanism analysis is presented and the mechanism of charge transfer between polymers and chromophores is experimentally and computationally validated. Thereafter, from an application perspective, multicolor cotton thread exhibition, ammonia gas detection, and information encryption are proposed. We showcase that color‐tunable RTP materials of a single phosphor in non‐aromatic polymers can be fabricated by merely charge transfer between polymers and chromophores, which are very appealing to the realm of multicolor display, information encryption, and gas detection.

## 2 Results and Discussion

Initially, a series of quinoline zwitterion (4‐X, X = Cl, Br, I, H, OH, CH_3_, OCH_3_, CHO) as chromophores were synthesized by nuclear reaction between 1, 3‐propyl sulphonolactone and quinolines with different substituents at the 4‐position (see details in Supporting Information). Especially, the structures of 4‐Cl and 4‐Br were characterized in detail by nuclear magnetic resonance (NMR) spectroscopy and high‐resolution mass spectrometry (HRMS), and their purity was checked by high‐performance liquid chromatography (HPLC) (Figures S1–S9, Supporting Information). Then, the chromophores 4‐X were doped with polyacrylamide (PAM) to construct RTP materials (PAM‐X) after vacuum drying at room temperature.

PAM‐Cl exhibits a green afterglow of 2–3 s after cessation of light excitation (Figure S10, Supporting Information), demonstrating the success in RTP materials synthesis by doping organic chromophores into polymers. From the photoluminescence spectra (Figure 1b; Figures S11a and S12a, Supporting Information), the high‐energy emission and the low‐energy emission of PAM‐Cl are centered at 404 and 520 nm, and the lifetimes reach 6.78 ns and 171.83 ms, respectively. With a delay of 1.0 ms, only the emission peak at 520 nm is detected (Figure 1b) and the peak is independent of excitation wavelength from the phosphorescence‐excitation mapping (Figure S12a, Supporting Information). Compared with the luminescence performance of 4‐Cl in methanol at 77 K (Figure 1c; Figure S13a, Supporting Information), PAM‐Cl shows similar phosphorescent emission, demonstrating that the phosphorescent peak of PAM‐Cl at 520 nm is attributed to isolated 4‐Cl molecules dispersed in the polymer matrix. To determine the optimal doping concentration of 4‐Cl in PAM, the lifetime (τ_P_) of the doped polymers with different molar ratios of the chromophore to monomer was measured. Results show that the optimal doping concentration of 4‐Cl is 0.1 mol% (Figure S14a, Supporting Information).

Then, 4‐Cl was doped into polyvinyl alcohol (PVA) with different alcoholysis degrees, or poly(N,N‐dimethylacrylamide) (PDMA) to evaluate the feasibility of other polymers. In agreement with the lifetimes, PAM‐Cl and PVA99%‐Cl emit bright green afterglows, while a faint phosphorescence or even no phosphorescence is observed for PVA74%‐Cl and PDMA‐Cl, respectively (Figure S10, Supporting Information). These results demonstrate that the abundant hydrogen bonds of polymers construct a rigid network to protect triplet exciton and suppress nonradiative decay, which has recently been reported for other PAM systems.^[^ ^23^ ^]^

**Figure 2:**
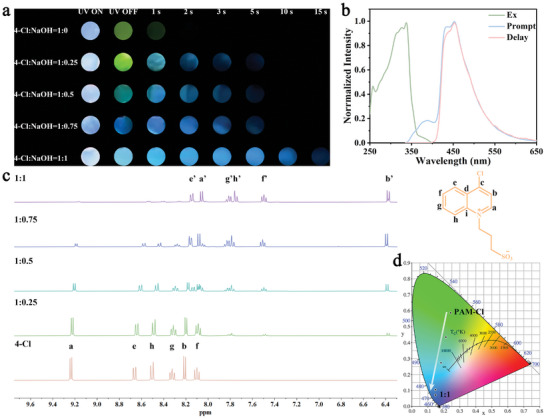
a) Afterglow images of PAM‐Cl‐NaOH with different equivalents of NaOH (*λ*_ex_ = 365 nm) after ceasing irradiation in air environment. b) Excitation spectrum, prompt and delayed phosphorescence spectra of 4‐Cl‐NaOH (1 ⨯ 10^‐6^ M) in methanol at 77 K. c) ^1^H NMR spectra (400 MHz, D_2_O) of 4‐Cl‐NaOH with addition of 0, 0.25, 0.5, 0.75, and 1 equivalent of NaOH. d) Chromaticity coordinate (CIE) of PAM‐Cl‐NaOH with 0, 0.25, 0.5, 0.75, and 1 equivalent of NaOH.

Interestingly, blue rather than green afterglow is observed for PAM‐Cl treated with alkali (PAM‐Cl‐NaOH) (**Figure** **2**a). To explain this behavior, the photophysical properties including phosphorescent emission and UV/vis absorption spectra of 4‐Cl‐NaOH were determined. Figure 2b shows that the phosphorescent emission of 4‐Cl‐NaOH in methanol at 77 K is basically consistent with that of PAM‐Cl‐NaOH (Figure 1b), further demonstrating that the phosphorescent emission of the polymer system is attributed to isolated chromophores. A newly formed UV/vis absorption peak is detected for 4‐Cl‐NaOH around 336 nm (Figure S15a, Supporting Information), compared with that of pure 4‐Cl (10^‐5^ mol L^−1^), which exhibits two distinct absorption bands around 238 and 320 nm assigned to π‐π* transition and intramolecular charge transfer (ICT), respectively. The newly formed absorption peak thereby reveals the changed electronic structure of 4‐Cl and the changed color of afterglow when treated with NaOH.

To get more insights into the effects of NaOH, we measured the luminescence performances of PAM‐Cl with different molar ratios of NaOH and 4‐Cl, setting the amount of 4‐Cl as 1.0 equivalent. Figure 1d depicts that a new emission band appears at 434 nm with the increase of NaOH content, whilst a remarkable time‐dependent emission is observed after ceasing the 365 nm UV lights. From the time‐resolved phosphorescence emission spectra (Figure 1d), two emission bands exist at 434 nm with a longer lifetime of 369.73 ms and 520 nm with a shorter lifetime of 221.1 ms upon the excitation at 340 nm, when the molar ratio of NaOH and 4‐Cl is 0.5:1. When NaOH content reaches above 0.75 equivalent, there is only one delayed emission peak at 434 nm left (Figure 1e). For the phosphorescence emission of PAM‐Cl and PAM‐Cl‐NaOH, τ_P_ prolongs from 171.83 ms to 602.67 ms, and Φ_P_ increases from 1.78% to 28.64%, respectively (Figure 1d,f; Figure S16a, Supporting Information). These results illustrate that the improvement of τ_P_ and Φ_P_ is achieved simultaneously.

We presume that the observed improvement of τ_P_ and Φ_P_ is related to the content of NaOH. This assumption is discussed with the help of ^1^H‐NMR titration experiments by gradually adding NaOH solution to 1.0 equivalent of 4‐Cl. Meanwhile, 4‐Cl‐NaOH was characterized in detail by ^1^H‐NMR, ^13^C‐NMR, HSQC, and HMBC (Figures S17–S20, Supporting Information). Indeed, as shown in Figure 2c, all ^1^H‐NMR peaks could be in one‐to‐one correspondence with the structural formula of 4‐Cl, suggesting that the molecular structure of the chromophores has not been broken by NaOH. However, all protons gradually shift to the high field upon the addition of NaOH from 0 to 1.0 equivalent because of the shielding effect, indicating the significant influence of NaOH in the electron cloud of the quinoline part. Notably, H_b_ moves from 8.09 to 6.19 ppm, demonstrating potential charge‐transfer interactions between H_b_ and hydroxide. C_b_ in ^13^C‐NMR also shifts to the high‐field region (Figures S3 and S18, Supporting Information), which agrees well with observations in the ^1^H‐NMR spectra. The CIE of PAM‐Cl with different NaOH contents is shown in Figure 2d, wherein the indicated colors change from green to blue in a linear manner.

Not coincidentally, PAM‐Br‐NaOH exhibits similar properties to PAM‐Cl‐NaOH (Figures S21–S28, Supporting Information). The delayed phosphorescence spectra of PAM‐Br and PAM‐Br‐NaOH at room temperature are consistent with that of 4‐Br and 4‐Br‐NaOH at 77K, respectively (Figures S26, Supporting Information). The introduction of NaOH shifts the delayed emission center of PAM‐Br from 530 to 434 nm, whilst increasing Φ_P_ from the original of 3.31% to 19.9% and prolonging τ_P_ from 12.31 to 589.31 ms. It is worth mentioning that a high‐field shift of protons is also observed for 4‐Br‐NaOH, especially for H_b_, which shifts from 8.29 to 6.20 ppm (Figure S28, Supporting Information).

According to the above results, the observed peak shift of the chromophores seems to be associated with their delayed emission bands. Therefore, 4‐hydroxyquinoline (QLOH) was selected as a chromophore to prepare PAM‐QLOH, which is weakly alkaline and could interact with acids. The excitation phosphorescence mapping shows that PAM‐QLOH undergoes a red shift from 445 to 475 nm with the addition of hydrogen ions (Figure S29, Supporting Information). Meanwhile, the aromatic protons of the quinoline all shift downfield. Both those observations illustrate the chemical shift of the chromophores is indeed related to their delayed emission spectra. To expand on this, polymeric RTP systems (PAM‐X) doped with quinoline zwitterion with other substituents (4‐X, X = I, H, OH, CH_3_, OCH_3_, CHO) were obtained. Similar results are also observed (Figure S30, Supporting Information), wherein H_b_ of 4‐X is sequentially arranged from upfield to downfield on ^1^H NMR spectra, and their delayed emission spectra and CIE are redshifted accordingly. In general, when the chromophores in this system show more electron deficiency, the low‐energy emission is more inclined to occur.

However, negligible change of excitation phosphorescence mapping is observed when 4‐H, 4‐CH_3_, chloroquinoline (QLCl), and bromoquinoline (QLBr) were doped into PAM with the addition of sufficient NaOH (Figure S12c–f, Supporting Information). Still, there is no significant shift in their UV/vis absorption spectra and ^1^H‐NMR titration results (Figures S15c–f; S31–S34, Supporting Information), further demonstrating strong charge‐transfer interactions only occur between electron‐deficiency chromophores and hydroxide.

Different from the luminescence performance upon alkali treatment, PAM‐Cl and PAM‐Br still show green afterglow when treated with acid. Compared with the photoluminescence spectra of the original PAM‐Cl (Figure 1b) and PAM‐Br (Figure S26a, Supporting Information), PAM‐Cl‐HCl and PAM‐Br‐HCl (Figure S35, Supporting Information) show enhanced phosphorescence emission intensity, indicating that after HCl is combined with the chromophores, Cl ion as an external heavy atom significantly promotes the spin‐orbit coupling and ISC for high RTP efficiency. Therefore, Φ_P_ increases from 1.76% to 1.79% for PAM‐Cl‐HCl and from 3.31% to 9.27% for PAM‐Br‐HCl, but τ_P_ decreases substantially for both PAM‐Cl‐HCl and PAM‐Br‐HCl. This evidence suggests that the improvement of τ_P_ and Φ_P_ simultaneously cannot be achieved simply by enhancing the ISC rate. According to Jablonski diagram, the key to achieving high Φ_P_ lies in enhancing intersystem crossing (ISC) rate (*k*_ISC_) from the lowest singlet state to triplet states, accelerating phosphorescent radiative rate (*k*_r_^P^) from the lowest triplet state to the ground state, and minimizing non‐radiative decay rate of phosphorescence (*k*_nr_^P^) and quenching rate (*k*_q_).^[^ ^24^ ^]^ However, to achieve long τ_P_, the rate constants of *k*_r_^P^, *k*_nr_^P^, and *k*_q_ should be reduced simultaneously. Paradoxically, a great *k*_r_^P^ needed to achieve high Φ_P_ will reduce τ_P_. Therefore, obtaining RTP materials with high Φ_P_ and long τ_P_ has been identified as a key challenge. In this context, working schemes have involved phosphine‐manipulated *p*‐π and π‐π synergy,^[^ ^25^ ^]^ matrices rigidification by multiple intermolecular interactions,^[^ ^26^ ^]^ and structural confinement via covalent crosslinking.^[^ ^27^ ^]^ The latter is maybe the most well‐known approach for polymeric RTP systems and can benefit from structural rigidification for phosphors and their environment, due to the cross‐linked nature of polymers. However, rather a complex process was required during the crosslinking. For example, stepwise taming of triplet excitons via multiple confinements has recently been proposed by Zhao and Yang as an interesting and efficient strategy for improving τ_P_ (more than 4 orders of magnitude) and Φ_P_ (16.04%) simultaneously.^[^ ^27a^ ^]^ Three‐level confinement was constructed including the primary confinement by copolymerization of 2‐vinyl naphthalene and vinyl acetate (VAc), secondary confinement by forming polyvinyl alcohol (PVA) hydrogen bonding networks via alcoholysis of PVAc, and tertiary confinement by crosslinking PVA networks with boric acid. This sophisticated structural confinement strategy is still far from being trivial. Therefore, this study achieved balanced Φ_P_ and τ_P_ using a charge transfer approach, representing an important achievement.

**Figure 3:**
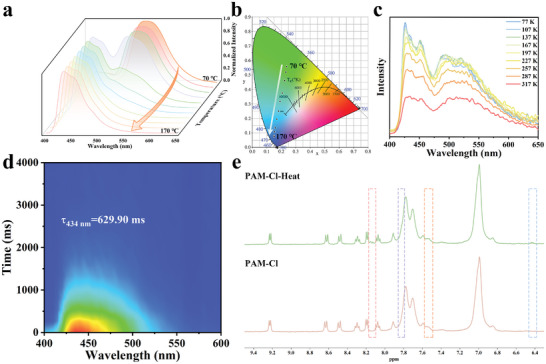
a) Normalized phosphorescence emission spectra of PAM‐Cl‐Heat treated at different temperatures for 10 min. b) CIE of PAM‐Cl‐Heat. c) Temperature‐dependent phosphorescence spectra of PAM‐Cl‐Heat treated at 110 °C for 10 min (excited at 340 nm, delayed time: 1.0 ms). d) Time‐resolved delayed spectra (excited at 340 nm, monitored at 434 nm) of PAM‐Cl‐Heat and its phosphorescence lifetime. e) ^1^H NMR spectra (400 MHz, D_2_O) of PAM‐Cl solution (down) and the solution after heating (up).

Surprisingly, the facile heat treatment also endows a blue afterglow to the polymeric RTP materials. PAM‐X were dried in a vacuum at room temperature and then heated separately from 70–170 °C with a gradient of 10 °C for 10 min to obtain PAM‐X‐Heat. With the increase in temperature, an afterglow with the longest duration time of 24 s is observed for PAM‐Cl‐Heat, shifting from green to cyan and finally to blue after the removal of 365 nm UV irradiation (Figure S36, Supporting Information). The delayed emission spectra clearly show the evolution of two emission bands after heat treatment (**Figure** **3**a). This behavior agrees well with the luminescence performances under alkali treatment (Figure 1e). A linear CIE is also observed with temperature increasing (Figure 3b). Identically, the new delayed emission band is also centered at 434 nm. To exclude the possibility of thermally activated delayed fluorescence (TADF), temperature‐variable delayed phosphorescence spectra were determined for PAM‐Cl‐Heat treated at 110 °C. The intensity of both two emission bands is enhanced progressively with the decrease in temperature, confirming the characteristics of phosphorescence (Figure 3c). Also, τ_P_ of PAM‐Cl‐Heat at 434 nm gradually decreases when the temperature rises from 77 K to 317 K (Figure S37, Supporting Information), owing to increased molecular motions of both 4‐Cl and PAM chains. These results imply that both delayed phosphorescence peaks originate from RTP emissive species and the new one has a higher triplet energy. The lifetime of PAM‐Cl‐Heat enhances from 171.83 ms at 520 nm to 629.90 ms at 434 nm (Figure 1, 3; Figure S14b, Supporting Information) and the corresponding Φ_P_ rises from 1.76% to 20.51% (Figure 1f).

Similar afterglow and delayed emission spectra are also observed for PAM‐Br‐Heat (Figures S38 and S39a, Supporting Information). Its τ_P_ reaches an astonishing fifty times the original from 12.31 ms to 613.58 ms (Figure S14c,d, Supporting Information) and Φ_P_ is up to 12.21%, which is 3.7 times that of PAM‐Br (Figure 1f). In brief, both PAM‐Cl‐Heat and PAM‐Br‐Heat achieve color‐tunable RTP with both long τ_P_ and high Φ_P_.

**Figure 4:**
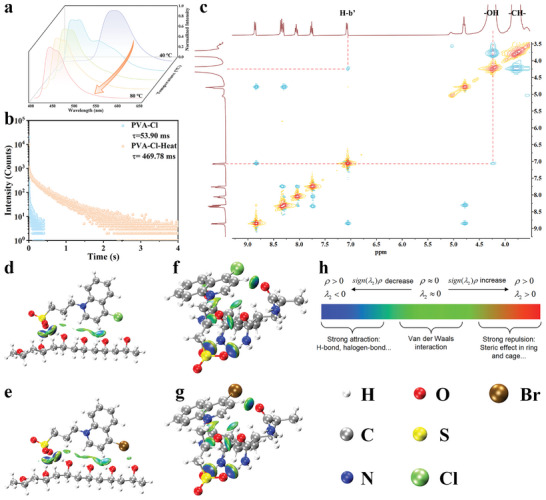
a) Normalized phosphorescence emission spectra of PVA‐Cl‐Heat treated at different temperatures for 10 min. b) Phosphorescence lifetime decay curves of PVA‐Cl (excited at 350 nm, monitored at 520 nm) and PVA‐Cl‐Heat (excited at 340 nm, monitored at 434 nm). c) 2D NOESY NMR spectrum (400 MHz, *d*_6_‐DMSO) of PVA and 4‐Cl after heating. The visualization of non‐covalent interactions of PVA‐Cl‐Heat d), PVA‐Br‐Heat e), PAM‐Cl‐Heat f) and PAM‐Br‐Heat g). h) The common interpretation of the coloring method of mapped function *sign(λ_2_)ρ* in the independent gradient model (IGM) and independent gradient model based on Hirshfeld partition (IGMH) maps.

Coincidentally, the formation of a new emission center after heat treatment is also observed for 4‐Cl or 4‐Br doped PVA with heat treatment (**Figure** **4**a; Figure S39b, Supporting Information). However, the photophysical properties of both PVA‐Cl‐Heat and PVA‐Br‐Heat are inferior to their PAM counterparts. For example, the τ_P_ is 469.78 ms and 410.31 ms at 434 nm (Figure 4b; Figure S39c, Supporting Information) and Φ_P_ is 8.04% and 4.72% for PVA‐Cl‐Heat and PVA‐Br‐Heat, respectively. The most striking difference is that the temperature at which the new emission center is formed is much lower in the case of PVA. It might be related to the molecular chain motility of polymers. We therefore quantify the glass transition temperature (*T*_g_) of the polymers by differential scanning calorimetry (DSC). Compared with PAM, PVA indeed has a lower *T*_g_ (Figure S40, Supporting Information), facilitating chain motility at lower temperatures.^[^ ^28^ ^]^

To further figure out the new emission center, ^1^H NMR spectra of PAM‐Cl and PAM‐Br with heat treatment were determined. It is found that the positions of new peaks are the same as those with alkali treatment (Figure 3e; Figure S39d, Supporting Information), indicating that both of their blue afterglow come from the same emission center. In the 2D NOESY spectra of AM and 4‐Cl/4‐Br with heat treatment (Figure S41), there is no correlation signal between ‐NH_2_ of AM and 4‐Cl/4‐Br, which is indicative of potential interactions between carbonyl groups and quinoline. In the 2D NOESY spectrum of PVA and 4‐Cl with heat treatment (Figure 4c), correlation signals between the protons on hydroxyl groups of PVA and H_b_’ of the quinoline moiety are observed, indicating the H‐O···π interactions between PVA and 4‐Cl. Similarly, the same correlation signals exist in the 2D NOESY spectrum of PVA and 4‐Br with heat treatment (Figure S39e, Supporting Information).

In the previous section, the observed alkali‐ and heat‐induced color‐tunable RTP is assumed related to the charge transfer between hydroxide or the electron‐rich polymers and the electron‐deficient chromophores. To verify the validity of this assumption, the electrostatic potential (ESP) distribution on the molecular surface of polymers and phosphors was mapped by density functional theory calculations (Figure S42a, Supporting Information). In brief, the quinoline rings of the original chromophores (4‐Cl and 4‐Br) show electron deficiency, and the new chromophores (4‐Cl‐NaOH and 4‐Br‐NaOH) are generated with an increase in electron density after combining with hydroxide. We note, at the same time, from the charge distribution of the polymer matrix that the electrons are concentrated on the carbonyl or hydroxyl groups. The distance between the hydrogen atoms of the chromophores and the electron‐rich groups of the polymers is calculated. As one can observe, the closest distance is the one between H_b_ and the electron‐rich groups (Figure S42b–e, Supporting Information), providing the possibility of the charge transfer between them. The independent gradient model (IGM) analysis is then applied to analyze the interactions between chromophores and PAM/PVA. The blue regions indicate stronger electrostatic attractions, whilst the green zones in the isosurface imply that the corresponding interactions are weak and may be regarded as Van der Waals interactions. Figure 4d–g shows that blue regions exist between H_b_ of quinoline and oxygen atoms of polymers, further illustrating that there are strong electrostatic interactions between H_b_ of the chromophores and the polymers.

**Figure 5:**
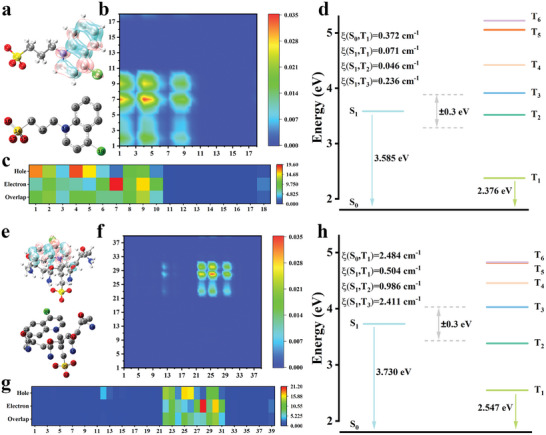
a) Electron/hole map of the excited state 2 of 4‐Cl (pink: electron; blue: hole) and the corresponding atomic number. b) Thermal maps corresponding to the atom‐atom charge transfer matrix of S_0_ → S_1_ of 4‐Cl. c) The heat map of atoms’ contribution to hole and electron of 4‐Cl. d) Diagrams of the TD‐DFT calculated energy levels and SOC constants of 4‐Cl. e) Electron/hole map of the excited state 2 of PAM‐Cl‐Heat (pink: electron; blue: hole) and the corresponding atomic number. f) Thermal maps corresponding to the atom‐atom charge transfer matrix of S_0_ → S_1_ of PAM‐Cl‐Heat. g) The heat map of atoms’ contribution to hole and electron of PAM‐Cl‐Heat. h) Diagrams of the TD‐DFT calculated energy levels and SOC constants of PAM‐Cl‐Heat.

To comprehend the different origins of the RTP from 4‐Cl and PAM‐Cl‐Heat, the wavefunctions of the excitation states were analyzed with a Multiwfn‐based time‐dependent density functional theory (TD‐DFT) calculation basis. Compared with the electron/hole map of 4‐Cl (**Figure** **5**a–c; Figures S43 and S44, Supporting Information), the electron and hole transitions of PAM‐Cl‐Heat obviously happen on the carbonyl of PAM and 4‐Cl (Figure 5e; Figures S45 and S46, Supporting Information). Meanwhile, both the thermal map (Figure 5f) corresponding to the atom‐atom charge transfer matrix and the heat map (Figure 5g) of atoms’ contribution to hole and electron show localization of partial holes on PAM, illustrating that the polymer matrix is involved in the charge transfer. It is found that the vertical excitation energy of S_1_ increases from 3.585 eV of 4‐Cl to 3.730 eV of PAM‐Cl‐Heat and the energy of T_1_ changes from 2.376 to 2.547 eV, respectively (Figure 5d,h). The calculated increased RTP transition energy gaps (T_1_–S_0_) would greatly enhance the ISC rate and phosphorescence quantum yield, which are consistent with the experimental data. Similar phenomena are also observed for PAM‐Br‐Heat, PVA‐Cl‐Heat and PVA‐Br‐Heat (Figures S47–S52, Supporting Information). The increased SOC is attributed to the heteroatoms of the polymers, including carbonyl groups of PAM and hydroxyl groups of PVA.

Analysis of rate constants further provides insight into the color‐tunable RTP mechanism (Table S1, Supporting Information). The sum of the low radiative rate constant (*k*_r_^P^) and the nonradiative decay rate of phosphorescence (*k*_nr_^P^) is of great importance for long‐lived emission according to Equation S1 in Table S1, Supporting Information. The change of *k*_r_^P^ is far less than that of *k*_nr_^P^, hinting at the long lifetime ascribing to the decreasing *k*_nr_^P^ value.^[^ ^29^ ^]^ The rate constants for ISC (*k*_ISC_) of PAM‐Cl (2.60 × 10^6^ s^−1^) is less than its radiative rate constant (*k*_r_^F^, 1.64 × 10^7^ s^−1^) of prompt fluorescence, resulting in the ISC process could not effectively compete with fluorescence radiation transition. However, both the *k*_ISC_ of PAM‐Cl‐NaOH (3.72 × 10^8^) and PAM‐Cl‐Heat (3.67 × 10^7^) is an order of magnitude larger than their *k*_r_^F^ values (3.66 × 10^7^ and 3.72 × 10^6^, respectively). These results indicate that PAM‐Cl‐NaOH and PAM‐Cl‐Heat are more inclined to produce phosphorescence, explaining why the low‐energy emission dominated the photoluminescence spectrum. Finally, all trends for rate constants are rather similar for alkali‐ and heat‐induced RTP materials, further suggesting the same charge transfer mechanism for alkali and heat treatment.

**Figure 6:**
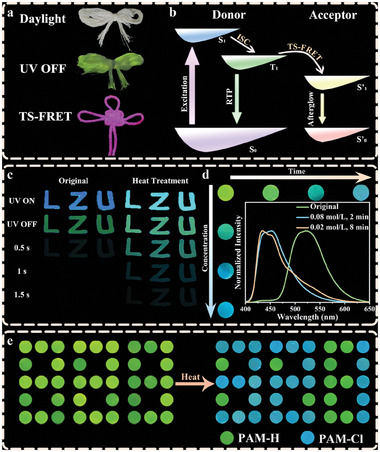
a) Photographs of cotton thread infiltrated with the solution of 4‐Cl as the dye solution under daylight and after removing 365 nm irradiation, and photographs of TS‐FRET between Rh‐B and 4‐Cl on cotton. b) Schematic diagram of the TS‐FRET process. c) Photographs of different letters on A4 paper by using the solution of 4‐Cl as the ink before and after heating. d) The afterglow images of PAM‐Cl films treated with ammonia gas at different concentrations (0.02, 0.04, 0.06, and 0.08 mol L^−1^) for 2 min or for different times (2, 4, 6, and 8 min) at 0.02 mol L^−1^. Inset is the normalized phosphorescence emission spectra of PAM‐Cl (Original), PAM‐Cl treated with 0.08 mol L^−1^ of ammonia gas for 2 min and 0.02 mol L^−1^ of ammonia gas for 8 min. e) Temperature‐induced anticounterfeiting realized by PAM‐Cl and PAM‐H.

The above results predict 4‐Cl doped polymers with hydrogen bonding can generate RTP. Therefore, we attempted to explore the optical properties of natural polymers such as cotton or paper, using 4‐Cl aqueous solution as a dye. As shown in **Figure** **6**a, the cotton thread infiltrated with the dye solution emits strong green phosphorescence after UV excitation. Since cotton is a highly available natural polymer that could be easily processed into clothing,^[^ ^30^ ^]^ the luminescence system presented herein could be used as anti‐counterfeited clothing. By introducing Rhodamine B (Rh‐B), whose excitation spectra are well overlapped with the delayed spectra of 4‐Cl (Figure S53, Supporting Information), a Chinese knot with a red afterglow is achieved by Förster resonance energy transfer (TS‐FRET) (Figure 6b). When writing the dye solution on A4 paper and drying at room temperature, the green afterglow of “LZU” is clearly observed after the excitation wavelength of 365 nm (Figure 6c). After heat treatment, the words emit blue afterglow with prolonged duration time, because of abundant hydroxyl and carbonyl groups existing in the paper.

The alkali‐ and heat‐induced color‐tunable RTP should open myriad possibilities in terms of functional applications. Here, several conceptual examples are proposed to demonstrate their potential applications in alkaline gas detection and information encryption. Freshly prepared PAM‐Cl films with a size of 1 × 1 cm were placed in ammonia gas with the concentration of 0.02, 0.04, 0.06, and 0.08 mol L^−1^ for 2 min, where the water in PAM‐Cl films reacted with ammonia gas to form hydroxide. As shown in Figure 6d, the phosphorescence shows a clear transition from green to blue. When the concentration was fixed at 0.02 mol L^−1^, the films exhibited a similar color gradient in ammonia gas for 2, 4, 6, and 8 min. Worth mentioning that the materials respond quickly (2 min) to high concentrations of ammonia (0.08 mol L^−1^). Even at low concentrations (0.02 mol L^−1^), ammonia gas could also lead to obvious changes of afterglow with prolonged time (8 min). Finally, digital encryption was determined using the number “888” formed with PAM‐Cl and PAM‐H. Green “888” is rendered after the removal of the UV light source. However, the real information (blue “321”) formed by PAM‐Cl appears after heating, since the phosphorescence of PAM‐H is independent of temperature (Figure 6e).

## 3 Conclusion

The emergence of a charge‐transfer‐mediated color‐tunable RTP system based on non‐aromatic polymers has been shown in this study. Since the key is simply doping electron‐deficient quinoline zwitterions into non‐aromatic polymers rich in hydrogen bonds, and no elaborate molecule design or complex fabrication process is needed, we believe this material will open new possibilities for wider use of polymeric color‐tunable RTP materials. The mechanism of charge transfer to produce new emission species between polymers and chromophores has been proposed and experimentally validated. Finally, the proposed color‐tunable RTP system shows ample applications in ammonia detection and information encryption. Even the quinoline zwitterions (4‐Cl) aqueous solution solely can be used as a dye to make cotton or paper phosphorescent. Such functions may be particularly applied in anti‐counterfeited clothing or writing, an application attracting increasing interest.

## Conflict of Interest

The authors declare no conflict of interest.

## Supporting information

Supporting Information

## Data Availability

The data that support the findings of this study are available from the corresponding author upon reasonable request.
