# Supplementary material for: Color‐Tunable Room‐Temperature Phosphorescence from Non‐Aromatic‐Polymer‐Involved Charge Transfer
Source: Adv Sci (Weinh). 2024 Jun 14;11(30):2404698. doi: 10.1002/advs.202404698 (PMC11321690; doi:10.1002/advs.202404698)
Supplement: Supplementary file 1 — Supporting Information [file ADVS-11-2404698-s001.pdf]

ADVANCED SCIENCE
Open Access

Supporting Information

for *Adv. Sci.*, DOI 10.1002/advs.202404698

Color-Tunable Room-Temperature Phosphorescence from Non-Aromatic-Polymer-Involved Charge Transfer

*Ningyan Li, Xipeng Yang, Binbin Wang, Panyi Chen, Yixian Ma, Qianqian Zhang, Yiyao Huang, Yan Zhang and Shaoyu Lü**

Supporting Information

Color-Tunable Room-Temperature Phosphorescence From Non-Aromatic-Polymer-Involved Charge Transfer

Ningyan Li,‡ Xipeng Yang,‡ Binbin Wang, Panyi Chen, Yixian Ma, Qianqian Zhang, Yiyao Huang, Yan Zhang, and Shaoyu Lü*

State Key Laboratory of Applied Organic Chemistry, Lanzhou Magnetic Resonance Center, Department of Chemistry and Chemical Engineering, Lanzhou University, Lanzhou 730000, China

*E-mail: lshy@lzu.edu.cn

‡ These authors contributed equally.

Contents

Materials····························S2

Characterization····························S2

Synthesis of 4-methoxyquinolin (QLOCH3) ····························S2

Synthesis of 4-iodoquinoline (QLI) ····························S3

Synthesis of quinoline zwitterion (4-X) ····························S3

Synthesis of the polymeric RTP systems····························S4

Computational details····························S4

Determination method of Φ_F and Φ_P····························S5

Statistical analysis····························S6

Figure S1-S52····························S7-S34

Table S1····························S35

References····························S35

Materials

Unless otherwise stated, all the materials used in the experiments, including 4-chloroquinoline (QLCl), 4-bromoquinoline (QLBr), 4-methylquinoline ($QLCH_3$), 4-hydroxyquinoline (QLH) and 1,3-propanesultone were purchased from commercial sources and used directly. Acrylamide (AM) and N,N-dimethylacrylamide (DMA) were purchased from J&K Scientific (Beijing, China). Polyvinyl alcohol (PVA, alcoholysis degree: 98%-99%) and PVA (alcoholysis degree: 72.5%-74.5%) were purchased from Aladdin (Shanghai, China).

Characterization

NMR spectra were all obtained with JEOL spectrometer (JNM-ECZ400S/L1 400 MHz) using D_2O or dimethyl sulfoxide-d_6 (DMSO-d_6) as solvents. High resolution mass spectrometry (HRMS) was determined on a Bruker APEX Ⅱ FT-MS mass spectrometer. High performance liquid chromatography (HPLC) was carried out by a Shimadzu LCMS-8060NX system using acetonitrile/carbinol (60/40, v/v) as the mobile phase. The flow rate was fixed at 2.0 mL/min and the injection volume was 5 μL. Absorption spectra of the chromophores were measured by an UV-vis spectrometer (UV-2500, Shimadzu). Differential scanning calorimeter (DSC) analysis was performed on a PerkinElmer instrument with a heating rate of 10 °C/min. The lifetime, time-resolved emission spectra, steady-state and delayed emission spectra (delay time: 1.0 ms), and temperature-dependent photoluminescence spectra were measured using an Edinburgh FLS 1000 fluorescence spectrophotometer equipped with a xenon lamp, laser device (330 nm), or a microsecond flash-lamp. The photoluminescence quantum yield was determined on an Edinburgh FLS 1000 fluorescence spectrophotometer with an integrating sphere.

Synthesis of 4-methoxyquinolin ($QLOCH_3$)

A solution of 4-hydroxyquinolin (6 mmol in MeOH, 7 ml, 1.0 equiv) was added to a solution of NaOMe (25wt% in MeOH, 5.0 equiv) at room temperature. The reaction mixture was stirred at 70 ^{o}C for 12 h and then was allowed to cool to 25 ^{o}C. After removing the solvent under reduced pressure, the residue was partitioned between ethyl acetate (10 ml) and water (10 ml). The organic layer was then washed with brine and was dried by adding sodium sulfate. The solution was filtered and evaporated to obtain the product 4-methoxyquinoline (Yield: 0.66 g, 76.6%). The synthetic route was shown in Scheme S1.

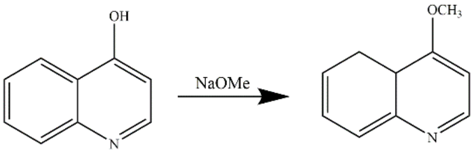

Scheme S1. The synthetic route of $QLOCH_3$.

Synthesis of 4-iodoquinoline (QLI)

A solution of 4-chloroquinoline (1 g in THF, 10 ml) was added to a solution of hydrochloric acid (8 mmol in 1,4-dioxane, 2 ml) at room temperature. After stirring for 5 min, the solvent was removed to obtain dry precipitate under reduced pressure. Anhydrous NaI (4 g) and hydrochloride were added to 30 ml anhydrous acetonitrile and refluxed for one day. A solution of 10 wt% K_2CO_3 and 5 wt% $NaHSO_3$ (10 mL) was prepared and mixed with the product at room temperature. Then, CH_2Cl_2 was used to extract the mixture. The organic phase was dried over anhydrous $MgSO_4$. After filtering, the solvent was evaporated under reduced pressure. The crude product residue was purified by chromatography on silica gel using hexane: EtOAc: Et_3N (100: 10: 1) as the mobile phase to collect the desired product (Yield: 0.9 g, 78.5%). ^1H NMR (400 MHz, $CDCl_3$): δ 8.44 (d, J = 4.6 Hz, 1H), 8.05-7.96 (m, 3H), 7.74 (ddd, J = 8.4, 6.9, 1.4 Hz, 1H), 7.61 (ddd, J = 8.3, 6.9, 1.3 Hz, 1H). The synthetic route was shown in Scheme S2.

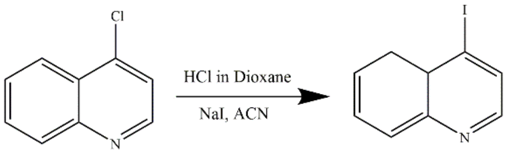

Scheme S2. The synthetic route of QLI.

Synthesis of quinoline zwitterion (4-X)

4-Chloroquinolin, 4-bromoquinolin, 4-iodoquinolin, 4-quinolin, 4-hydroxyquinolin, 4-methylquinolin, 4-methoxyquinolin or 4-formylquinolin (5 mmol) was thoroughly mixed with 1,3-propane sultone (6 mmol). The mixture was reacted in an oil bath at 130 ^oC for 2-4 h. After cooling to room temperature, the products were washed with diethyl ether and recrystallized from diluted methanol. The synthetic route of quinoline zwitterion (4-X) was shown in Scheme S3.

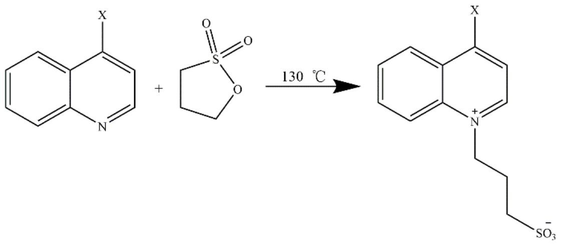

Scheme S3. The synthetic route of 4-X.

3-(4-Chloroquinolin-1-ium-1-yl)propane-1-sulfonate (4-Cl): Yield: 88.7%. ^{1}H NMR (400 MHz, D_2O): δ 9.24 (d, *J* = 6.5 Hz, 1H), 8.66 (dd, *J* = 8.7, 1.4 Hz, 1H), 8.51 (d, *J* = 8.9 Hz, 1H), 8.32 (ddd, *J* = 8.8, 7.1, 1.5 Hz, 1H), 8.21 (d, *J* = 6.4 Hz, 1H), 8.14-8.06 (m, 1H), 5.24-5.15 (m, 2H), 3.10 (t, *J* = 7.1 Hz, 2H), 2.54 (dq, *J* = 9.5, 7.3 Hz, 2H). HRMS m/z: $[M+H]^+$ calcd for $C_{12}H_{13}ClNO_3S$, 286.0299; found 286.0301.

3-(4-Bromoquinolin-1-ium-1-yl)propane-1-sulfonate (4-Br): Yield: 77.6%. ^{1}H NMR (400 MHz, D_2O): δ 9.06 (d, *J* = 6.4 Hz, 1H), 8.57 (dd, *J* = 8.7, 1.5 Hz, 1H), 8.43 (d, *J* = 9.0 Hz, 1H), 8.38 (s, 0H), 8.26 (ddd, *J* = 8.7, 6.9, 1.5 Hz, 1H), 8.07-7.99 (m, 1H), 5.17- 5.09 (m, 2H), 3.05 (t, *J* = 7.1 Hz, 2H), 2.49 (dq, *J* = 9.6, 7.3 Hz, 2H). HRMS m/z: $[M+H]^+$ calcd for $C_{12}H_{13}BrNO_3S$, 329.9794; found 329.9796.

3-(4-Iodoquinolin-1-ium-1-yl)propane-1-sulfonate (4-I): Yield: 52.8%. ^{1}H NMR (400 MHz, D_2O): δ 8.75 (d, *J* = 6.8 Hz, 1H), 8.32-8.24 (m, 1H), 8.00-7.86 (m, 2H), 7.71 (ddd, *J* = 8.2, 6.8, 1.3 Hz, 1H), 7.26 (d, *J* = 6.8 Hz, 1H), 4.58 (t, *J* = 6.1 Hz, 2H), 3.21 -3.13 (m, 2H), 2.46-2.35 (m, 2H).

3-(4-Quinolin-1-ium-1-yl)propane-1-sulfonate (4-H): Yield: 91.2%. ^{1}H NMR (400 MHz, D_2O): δ 9.23 (dd, *J* = 5.9, 1.4 Hz, 1H), 9.07 (d, *J* = 8.3 Hz, 1H), 8.43 (d, *J* = 9.1 Hz, 1H), 8.31 (dd, *J* = 8.2, 1.5 Hz, 1H), 8.21 (ddd, *J* = 8.9, 7.0, 1.5 Hz, 1H), 8.03-7.91 (m, 2H), 5.21-5.11 (m, 2H), 3.05 (t, *J* = 7.1 Hz, 2H), 2.50 (dq, *J* = 9.5, 7.3 Hz, 2H).

3-(4-Hydroxyquinolin-1-ium-1-yl)propane-1-sulfonate (4-OH): Yield: 59.3%. ^{1}H NMR (400 MHz, D_2O): δ 8.81 (d, *J* = 6.8 Hz, 1H), 8.36 (dd, *J* = 8.7, 1.4 Hz, 1H), 8.06-7.93 (m, 2H), 7.77 (ddd, *J* = 8.3, 6.9, 1.4 Hz, 1H), 7.40-7.29 (m, 1H), 4.64 (t, *J* = 6.1 Hz, 2H), 3.25-3.17 (m, 2H), 2.50-2.39 (m, 2H).

3-(4-Methylquinolin-1-ium-1-yl)propane-1-sulfonate (4-CH_3): Yield: 81.4%. ^{1}H NMR (400 MHz, D_2O): δ 9.02 (d, *J* = 6.1 Hz, 1H), 8.42 (dd, *J* = 8.4, 1.4 Hz, 1H), 8.37 (d, *J* = 9.0 Hz, 1H), 8.16 (ddd, *J* = 9.0, 7.2, 1.5 Hz, 1H), 7.94 (dd, *J* = 8.5, 7.1 Hz, 1H), 7.83 (d, *J* = 6.2 Hz, 1H), 5.12-5.04 (m, 2H), 3.02 (t, *J* = 7.1 Hz, 2H), 2.46 (p, *J* = 7.4 Hz, 2H).

3-(4-Methoxyquinolin-1-ium-1-yl)propane-1-sulfonate (4-OCH_3): Yield: 73.9%. ^{1}H NMR (400 MHz, D_2O): δ 8.98 (d, *J* = 7.0 Hz, 1H), 8.45 (dd, *J* = 8.4, 1.5 Hz, 1H), 8.22 (d, *J* = 8.8 Hz, 1H), 8.10 (ddd, *J* = 8.8, 7.0, 1.5 Hz, 1H), 7.86-7.78 (m, 1H), 7.35 (d, *J* = 7.1 Hz, 1H), 4.93 (t, *J* = 7.6 Hz, 2H), 3.01 (t, *J* = 7.2 Hz, 2H), 2.48-2.36 (m, 2H).

3-(4-Formylquinolin-1-ium-1-yl)propane-1-sulfonate (4-CHO): Yield: 63.1%. ^{1}H NMR (400 MHz, D_2O): δ 9.30 (d, *J* = 6.0 Hz, 1H), 8.61 (d, *J* = 8.7 Hz, 1H), 8.47 (d, *J* = 9.0 Hz,

1H), 8.22 (dd, *J* = 9.2, 6.8 Hz, 2H), 7.99 (t, *J* = 7.8 Hz, 1H), 6.82 (s, 1H), 5.23-5.14 (m, 2H), 3.06 (t, *J* = 7.1 Hz, 2H), 2.51 (p, *J* = 7.3 Hz, 2H).

Synthesis of the polymeric RTP systems

PAM-X: $K_2S_2O_8$ (1 mg), N,N-dimethylacrylamide (1 mg), acrylamide (0.4073 g) and 4-X (1.6 mg) were dissolved in deionized water (1 ml) and injected into a mold of 1 mm. The mixture reacted at 50 °C for 2 h. The product was dried in vacuum at room temperature to obtain PAM-X.

PAM-X-NaOH: $K_2S_2O_8$ (1 mg), N,N-dimethylacrylamide (1 mg), acrylamide (0.4073 g) and 4-X (1.6 mg) were dissolved in 0.006 M NaOH solution (1 ml) and injected into a mold of 1 mm. The mixture reacted at 50 °C for 2 h. The product was dried in vacuum at room temperature to obtain PAM-X-NaOH.

PAM-X-Heat: PAM-X was heated at 170 °C for 10 min to obtain PAM-X-Heat.

PVA-X: PVA (1 g) was dissolved in deionized water (9 g) at 90 °C. After the solution was cooled to room temperature, an aqueous solution of 4-X (6.4 mg) was added. The mixture was dried in vacuum at room temperature to obtain PVA-X.

PVA-X-NaOH: PVA (1 g) was dissolved in deionized water (9 g) at 90 °C. After the solution was cooled to room temperature, an aqueous solution of 4-X (6.4 mg) and 0.023 M NaOH solution (1 ml) was added. The mixture was dried in vacuum at room temperature to obtain PVA-X-NaOH.

PVA-X-Heat: PVA-X was heated at 80 °C for 10 min to obtain PVA-X-Heat.

Computational details

In this work, on the basis of Gaussian 16 C.02[1] packages, all the sample structures were fully optimized by M06-2X[2] with def2-SVP[3] basis set. The vertical excitation energies were calculated based on the optimized ground state geometries (S_0). In our system, the hole-electron analyzation method was adopted to study the excitation characters, through wave function analysis program Multiwfn 3.8 dev[4]. The hole-electron distribution map and the molecular orbitals were both visualized through VMD 1.9.3[5]. Intersystem crossing (ISC) process of the sample structures was further evaluated through ORCA 5.0.4[6] package with M06-2X[2] and def2-TZVP[3, 7] basic set. The electronic structures and the spin-orbit coupling (SOC) matrix elements between singlet and triplet states of the sample structures were obtained on basis of optimized geometries in S_1 excited state.

Determination method of Φ_F and Φ_P

The peak fitting method has been widely used to calculate the fluorescence and phosphorescence quantum yields according to precious literatures [8]. The fluorescence and phosphorescence emission bands could be separated in the steady-state PL spectra and their integral areas could be obtained. As shown in Scheme S4, A_1 and A_2 represent the integral areas of fluorescence and phosphorescence, respectively. Therefore, the fluorescent and phosphorescent quantum yields (Φ_F and Φ_P) were calculated by:

$$\Phi_F = \Phi \frac{A_1}{A_1+A_2}$$

$$\Phi_P = \Phi \frac{A_2}{A_1+A_2}$$

where Φ refers to the absolute photoluminescence quantum yield measured using Edinburgh FL1000 spectrometer.

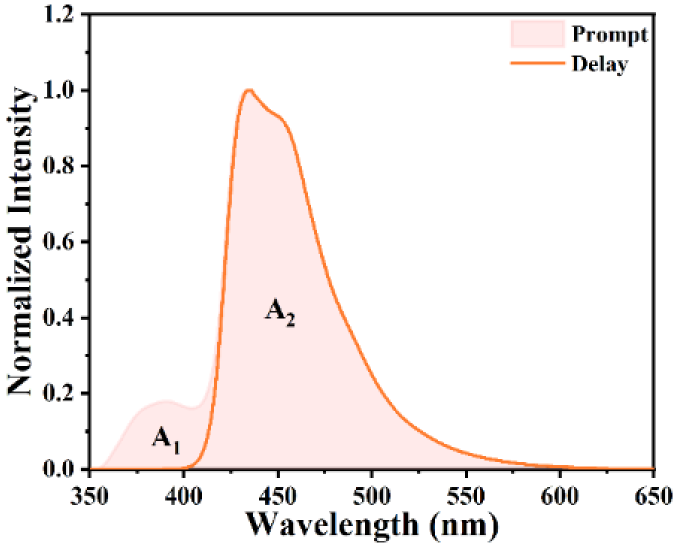

Scheme S4. Peak-differentation-imitating analysis of PL spectrum.

Statistical analysis

All of photoluminescence quantum yields were measured and calculated from three parallel samples to obtain the average value. The normalized photoluminescence spectra were carried out by setting the intensity at the peak maximum as 1 using the Origin software. The phosphorescence lifetimes were fitted by the exponential fitting function of Fluoracle software, keeping the χ^2 in the range from 0.9 to 1.3.

Figures

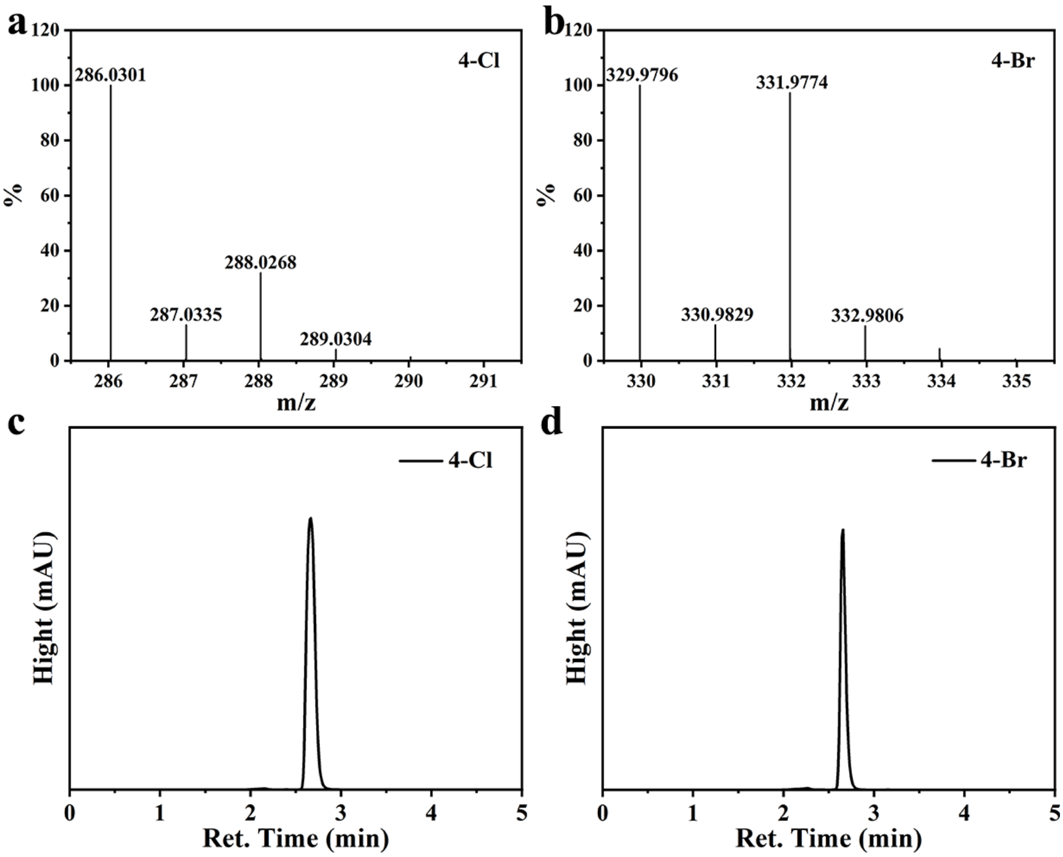

Figure S1. HRMS spectrum of 4-Cl (a) and 4-Br (b); HPLC spectrum of 4-Cl (c) and 4-Br (d).

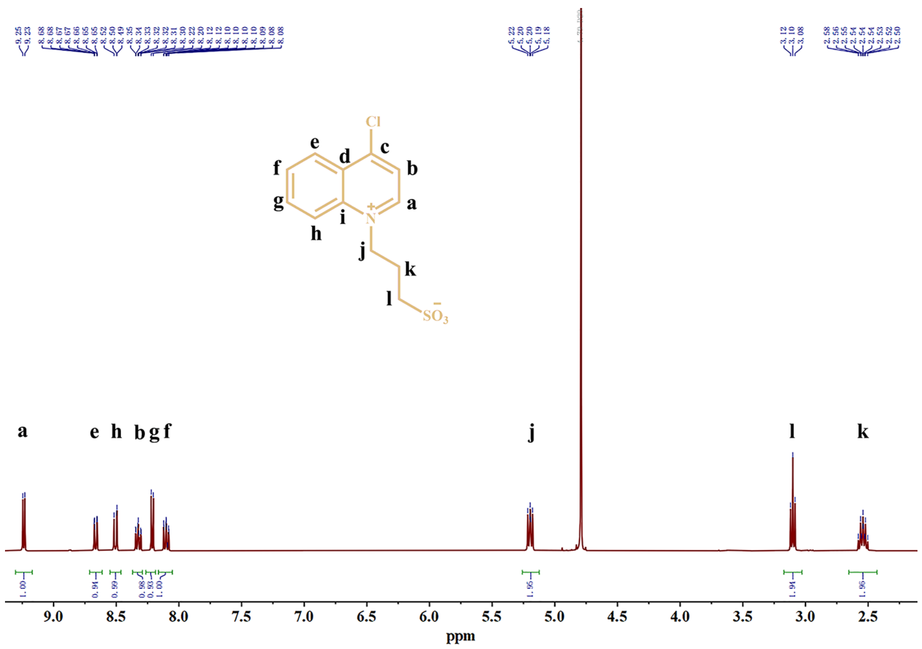

Figure S2. ¹H NMR (400 MHz, D2O) spectrum of 4-Cl.

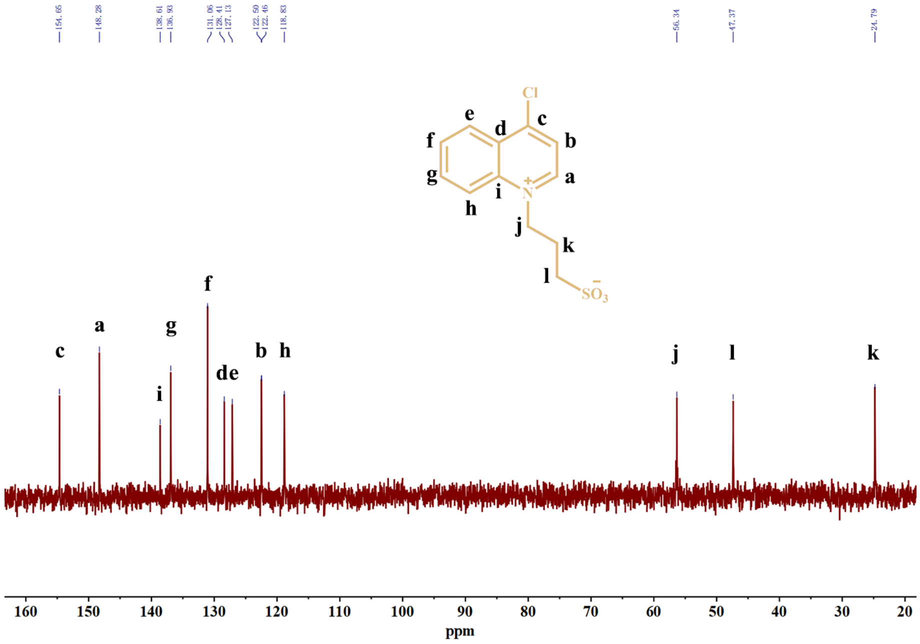

Figure S3. ^{13}C NMR (400 MHz, D_2O) spectrum of 4-Cl.

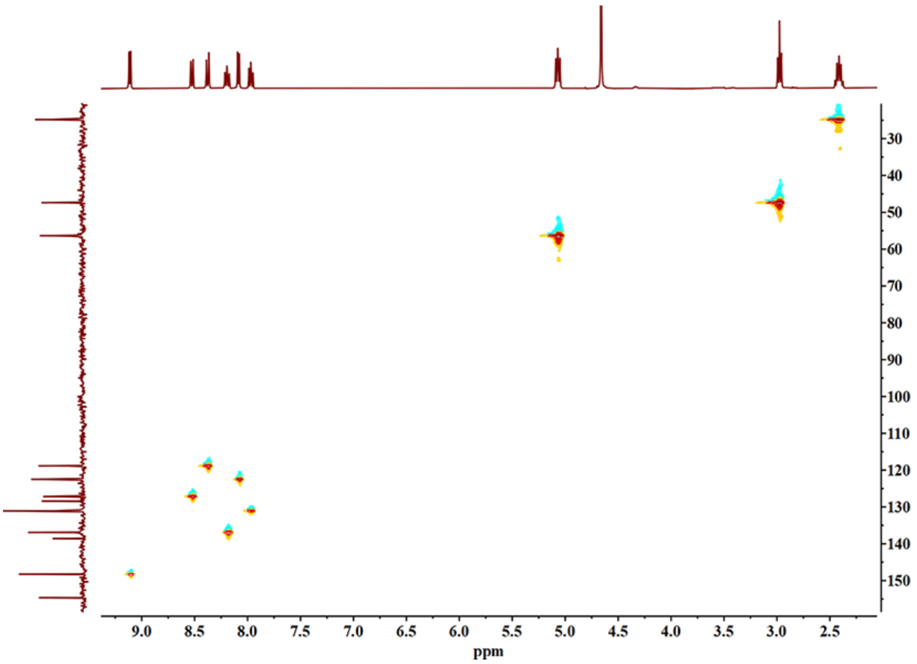

Figure S4. HSQC (400 MHz, D_2O) spectrum of 4-Cl.

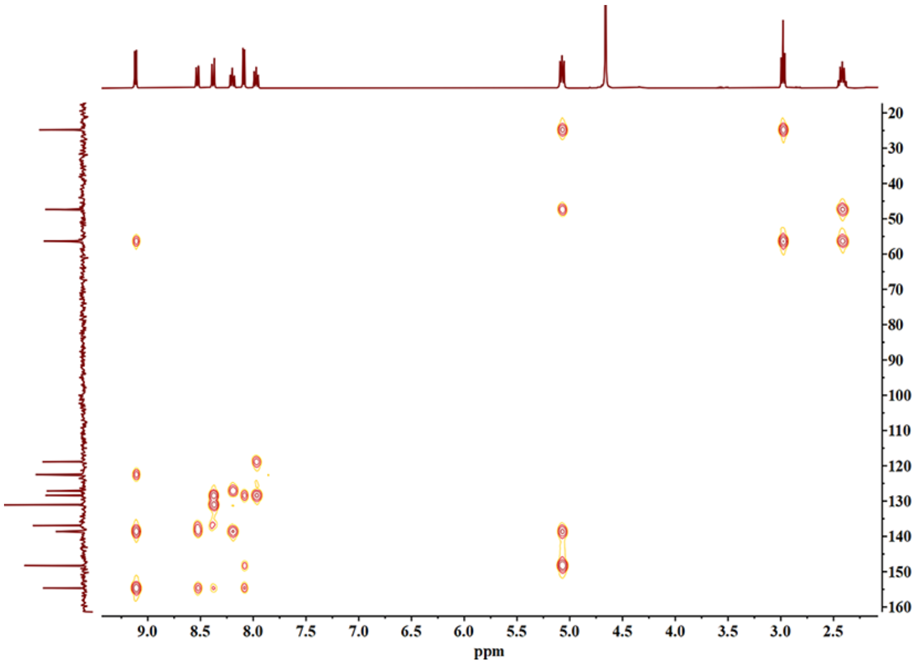

Figure S5. HMBC (400 MHz, D_2O) spectrum of 4-Cl.

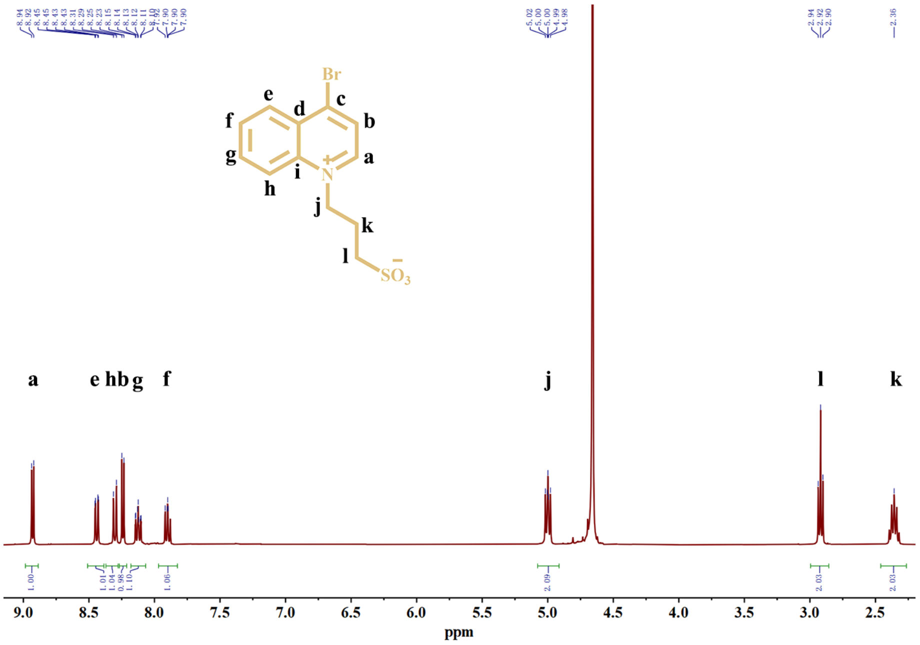

Figure S6. ^1H NMR (400 MHz, D_2O) spectrum of 4-Br.

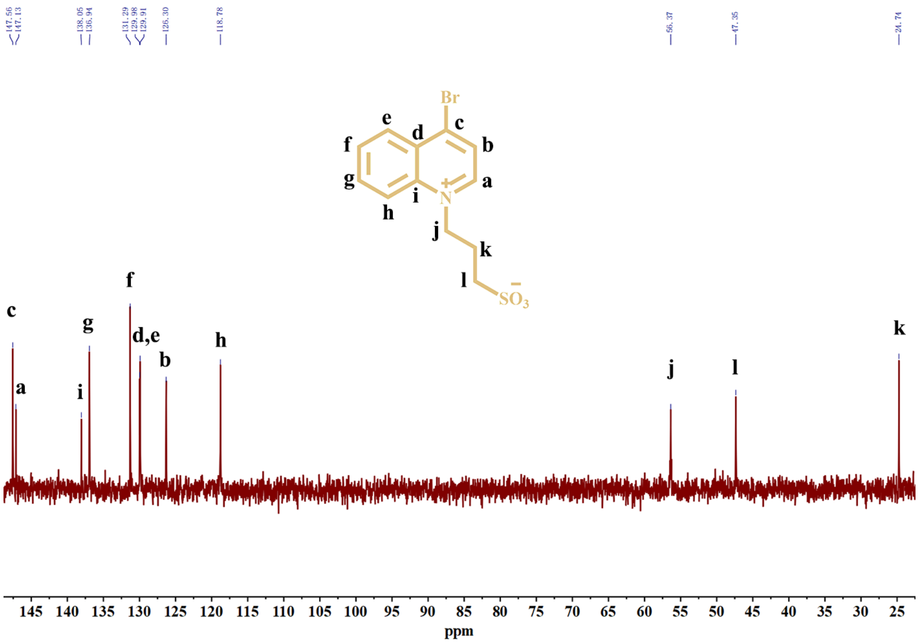

Figure S7. ^{13}C NMR (400 MHz, D_2O) spectrum of 4-Br.

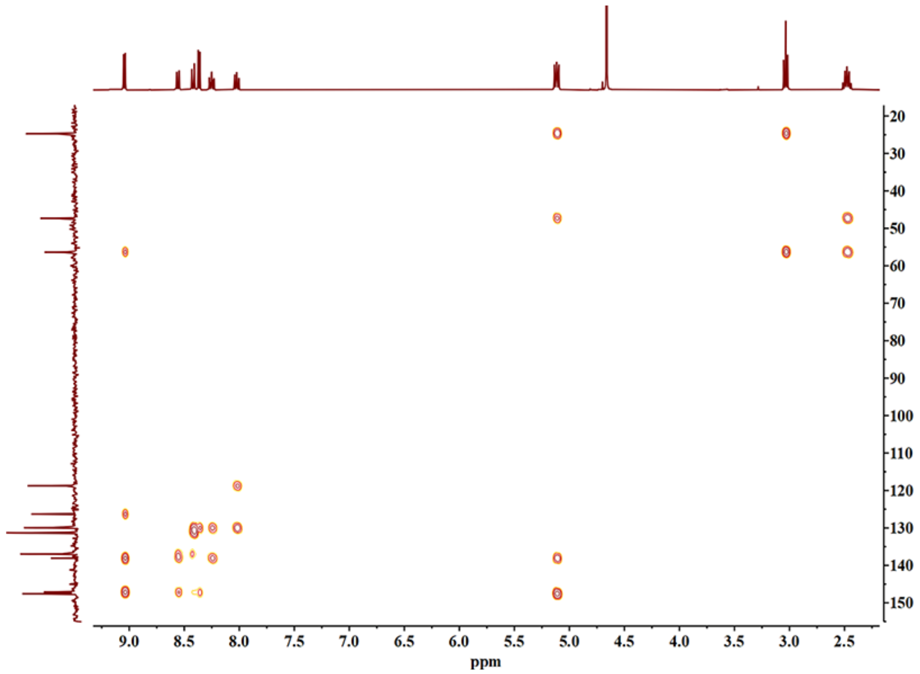

Figure S8. HMBC (400 MHz, D_2O) spectrum of 4-Br.

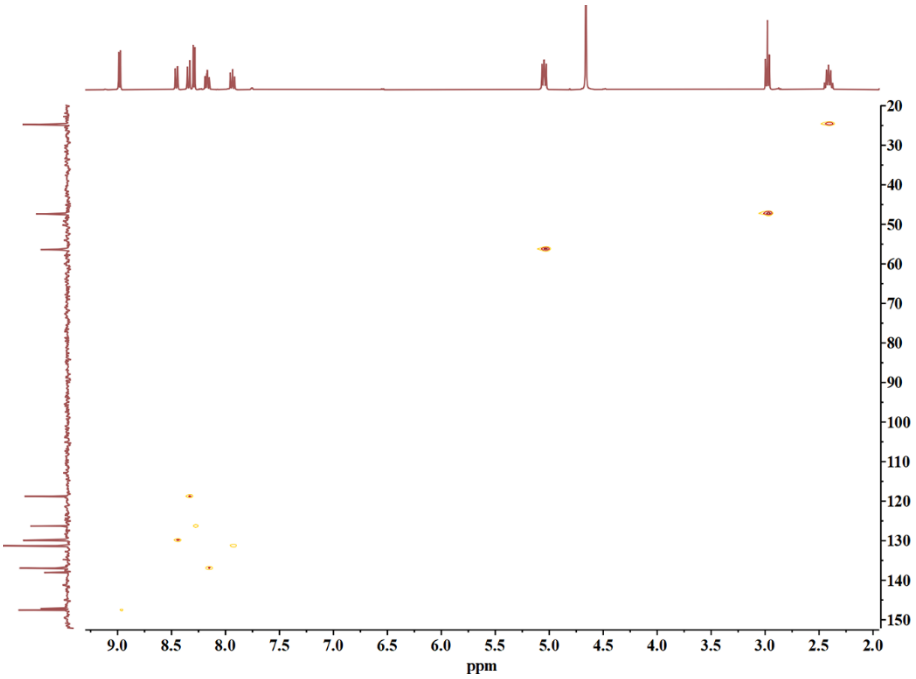

Figure S9. HSQC (400 MHz, D_2O) spectrum of 4-Br.

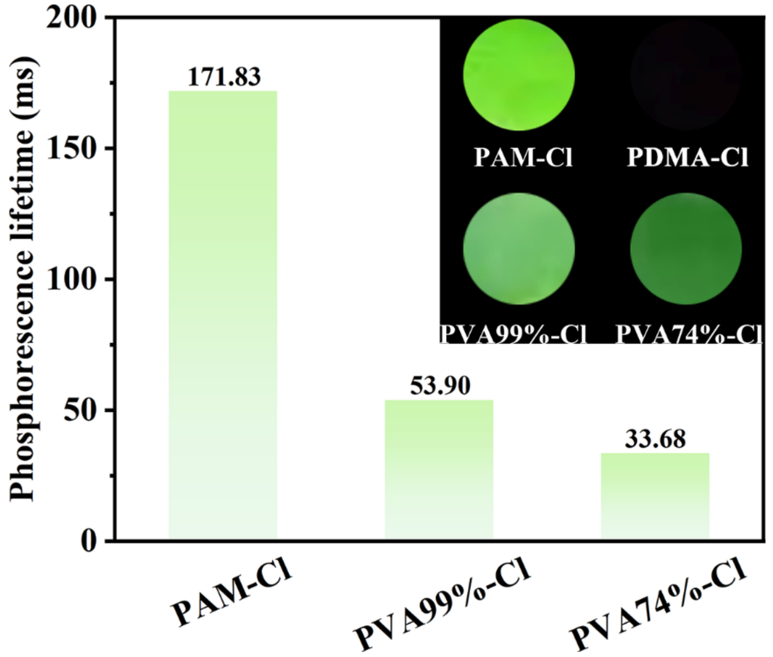

Figure S10. Phosphorescence lifetime of PAM-Cl, PVA99%-Cl, and PVA74%-Cl and their afterglow images. No phosphorescence was detected for PDMA-Cl.

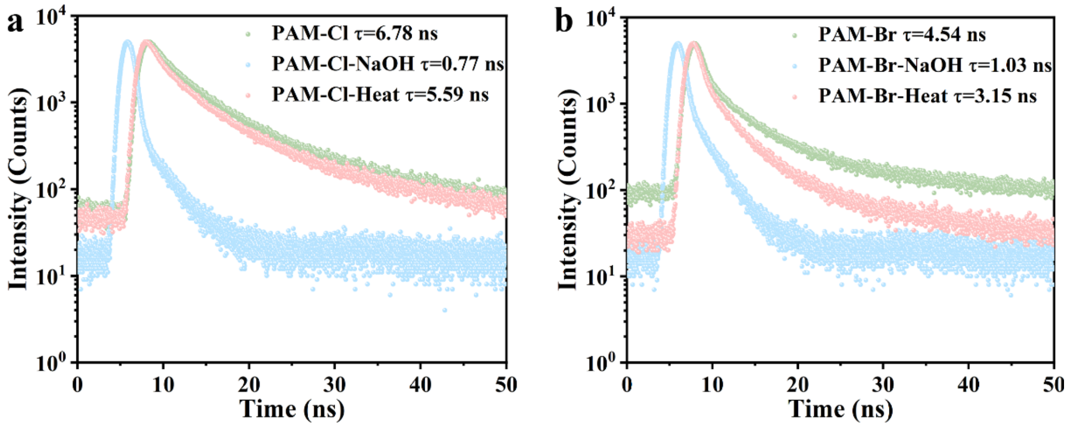

Figure S11. (a) PL decay of PAM-Cl, PAM-Cl-NaOH and PAM-Cl-Heat at room temperature. (b) PL decay of PAM-Br, PAM-Br-NaOH and PAM-Br-Heat at room temperature.

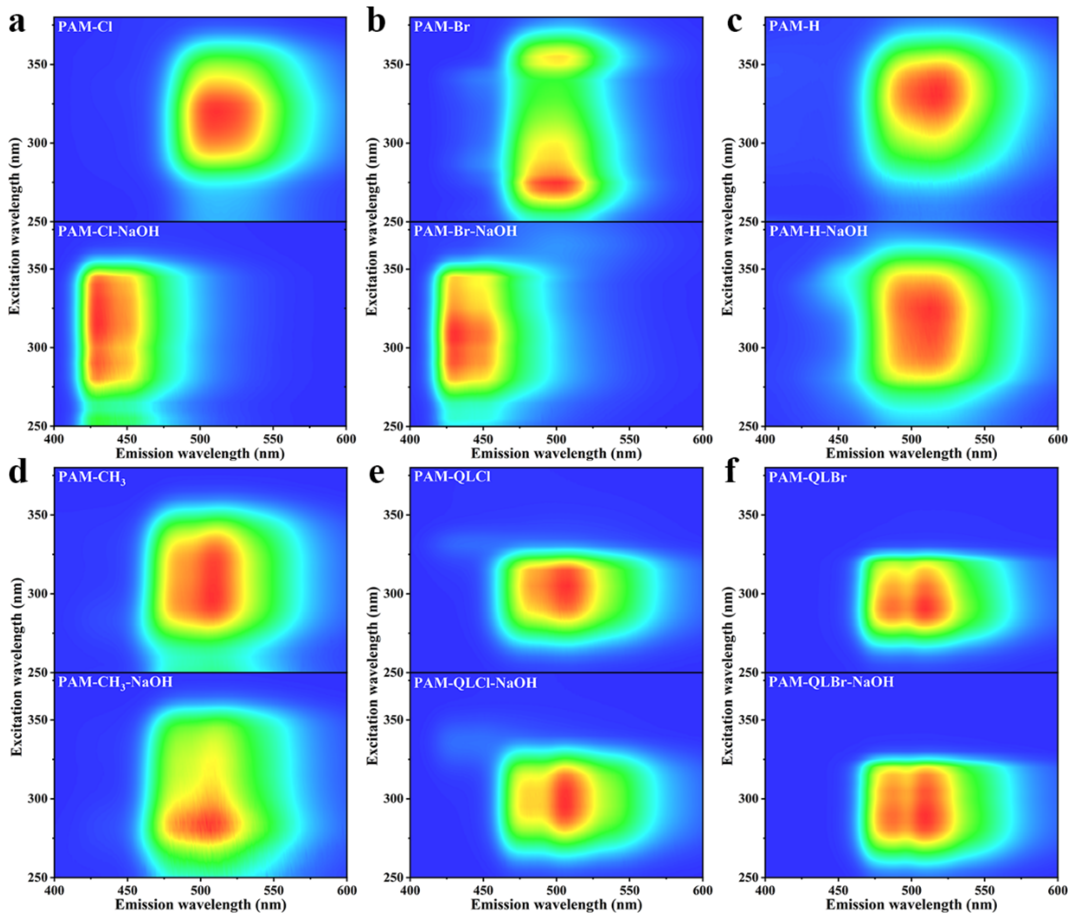

Figure S12. Phosphorescence-excitation mapping of PAM-Cl and PAM-Cl-NaOH (a), PAM-Br and PAM-Br-NaOH (b), PAM-H and PAM-H-NaOH (c), PAM-CH_3 and PAM-CH_3-NaOH (d), PAM-QLCl and PAM-QLCl-NaOH (e), PAM-QLBr and PAM-QLBr-NaOH (f).

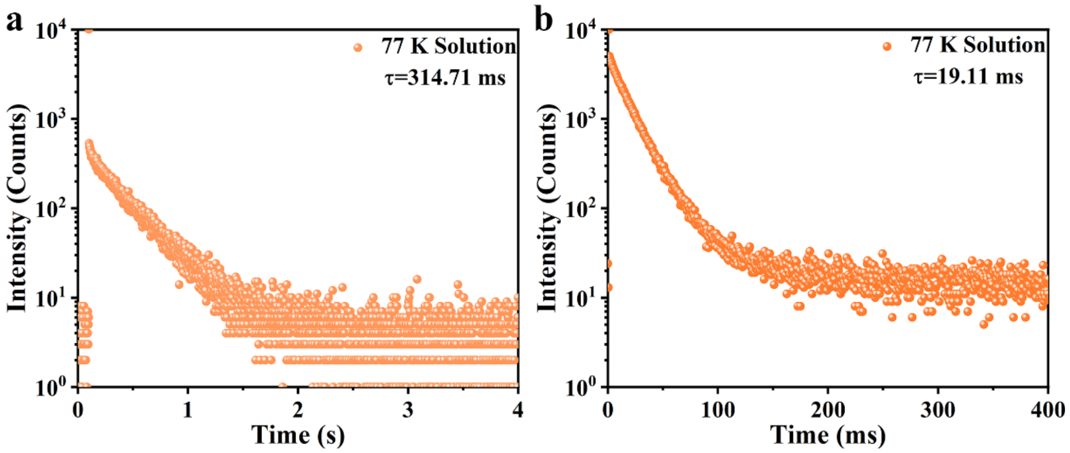

Figure S13. Phosphorescence lifetime decay curves of 4-Cl in methanol (a) and 4-Br in methanol (b) at 77 K (C = 1×10^{-6} M, excited at 350 nm, monitored at 520 nm).

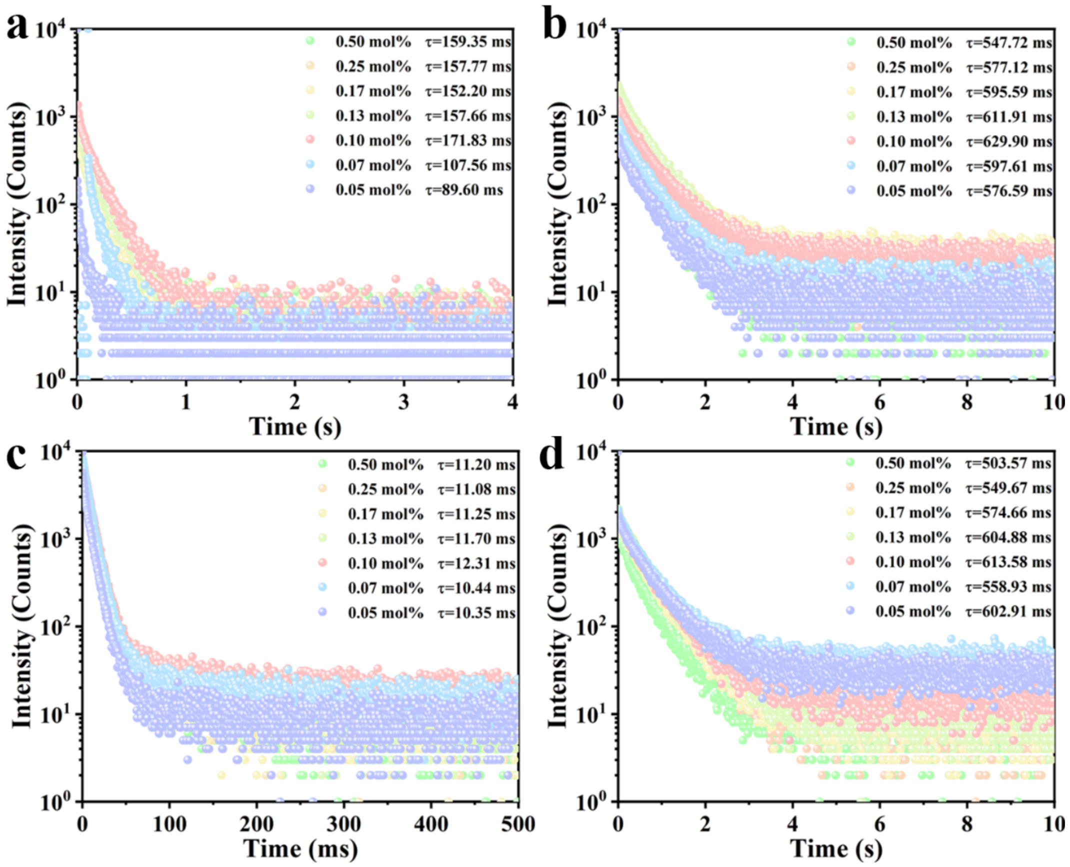

Figure S14. Phosphorescence lifetime of PAM-Cl (a, excited at 340 nm, monitored at 520 nm), PAM-Cl-Heat (b, excited at 340 nm, monitored at 434 nm), PAM-Br (c, excited at 350 nm, monitored at 530 nm), and PAM-Br-Heat (d, excited at 340 nm, monitored at 434 nm) at different concentrations.

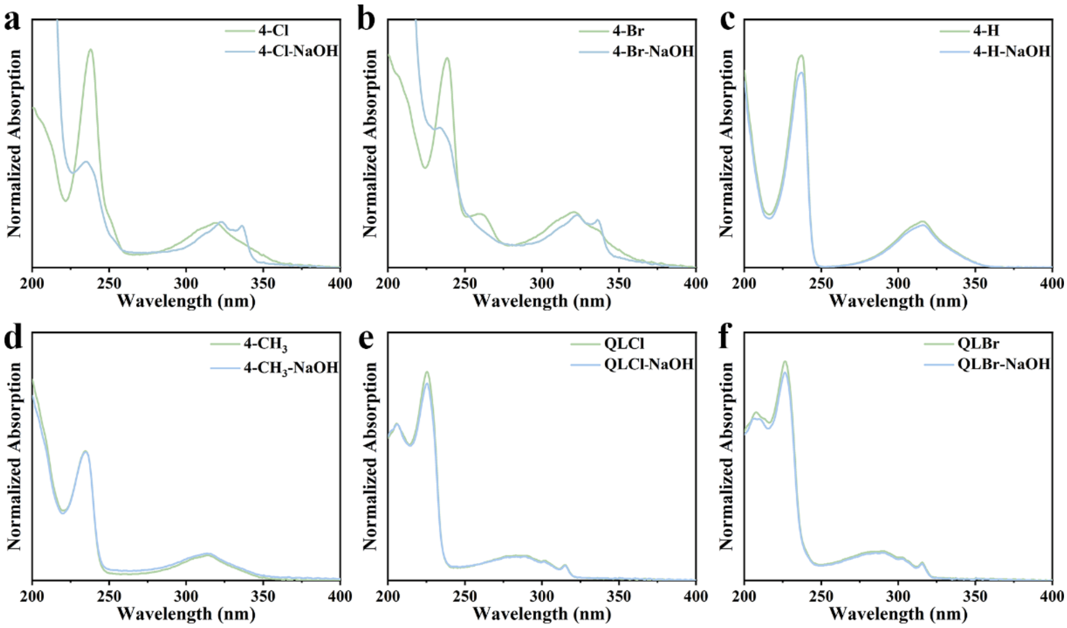

Figure S15. Normalized UV-vis absorbance spectra of 4-Cl and 4-Cl-NaOH (a), 4-Br and 4-Br-NaOH (b), 4-H and 4-H-NaOH (c), 4-CH_3 and 4-CH_3-NaOH (d), QLCl and QLCl-NaOH (e), QLBr and QLBr-NaOH (f).

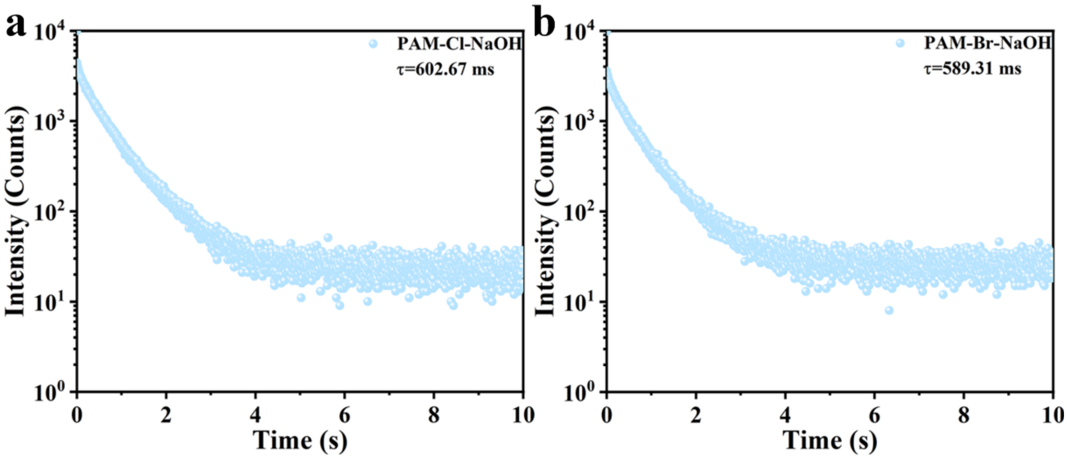

Figure S16. Phosphorescence lifetime decay curves of PAM-Cl-NaOH (a) and PAM-Br-NaOH (b) (excited at 340 nm, monitored at 434 nm).

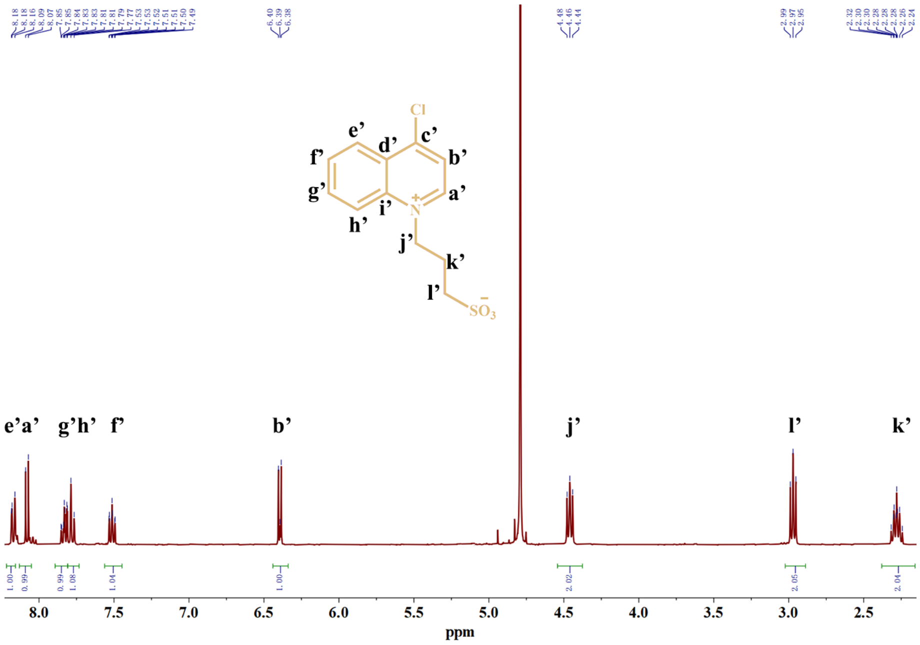

Figure S17. ^{1}H NMR (400 MHz, D_2O) spectrum of 4-Cl-NaOH.

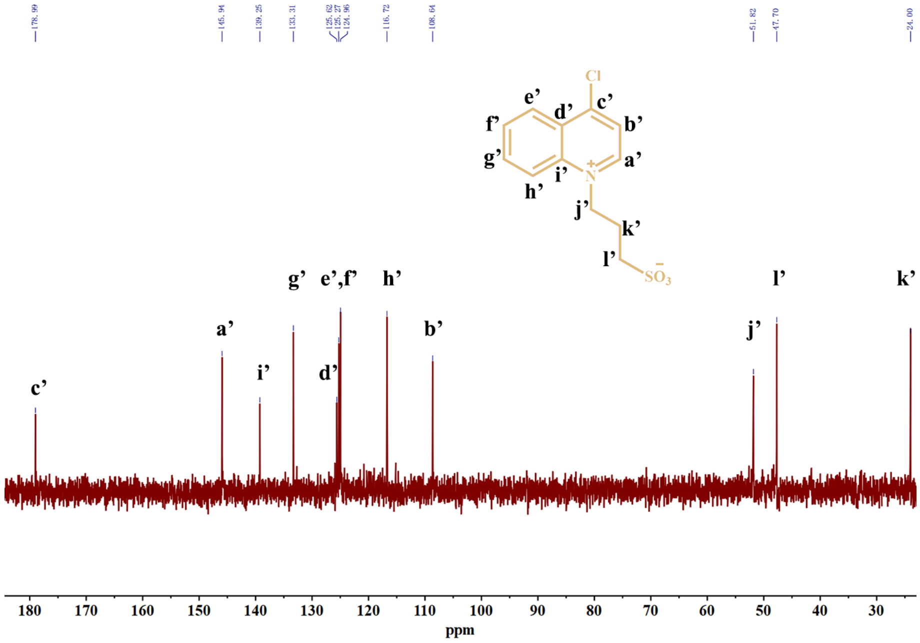

Figure S18. ^{13}C NMR (400 MHz, D_2O) spectrum of 4-Cl-NaOH.

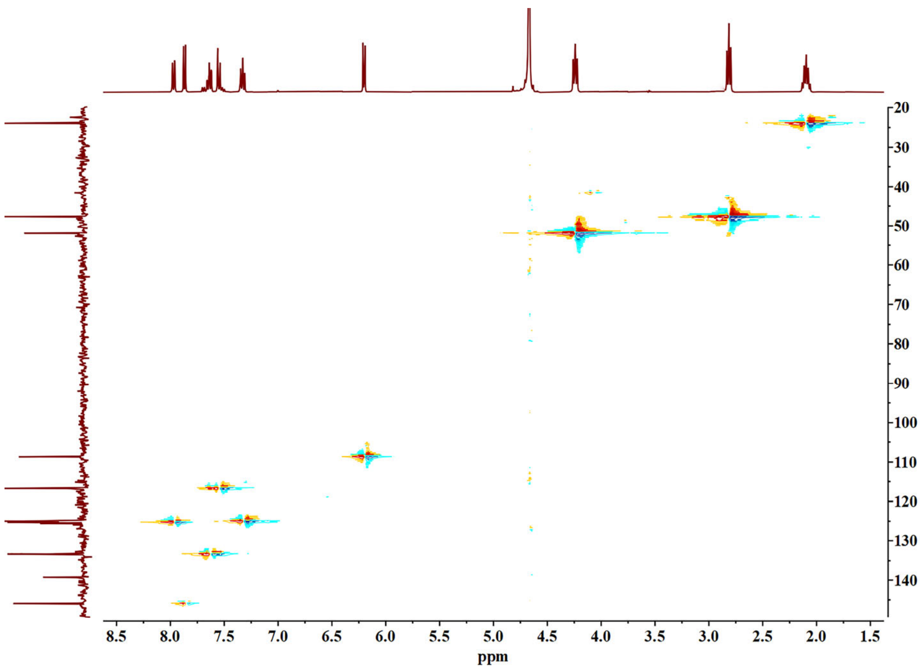

Figure S19. HSQC (400 MHz, D_2O) spectrum of 4-Cl-NaOH.

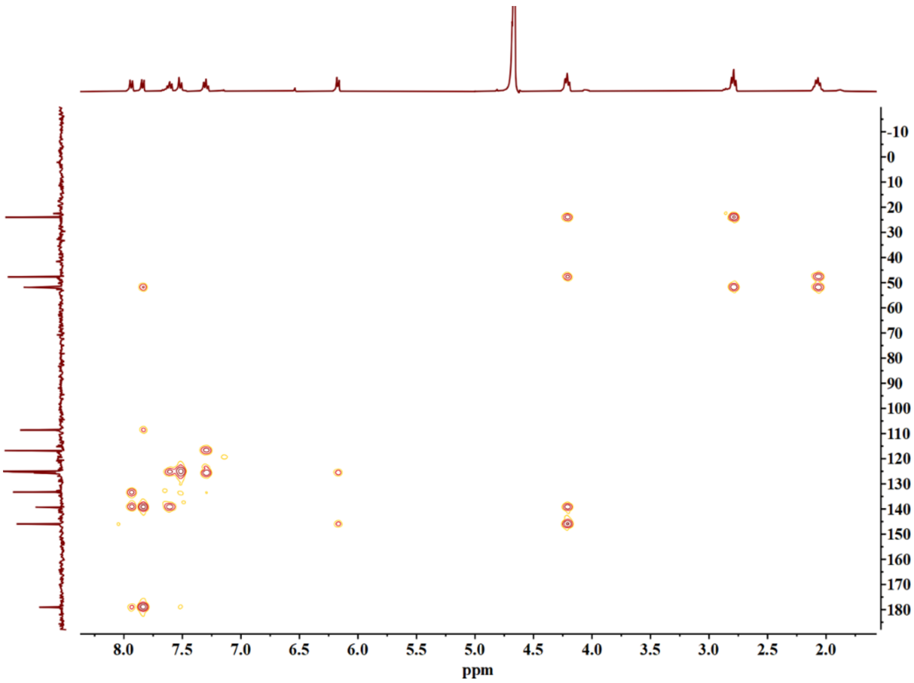

Figure S20. HMBC (400 MHz, D_2O) spectrum of 4-Cl-NaOH.

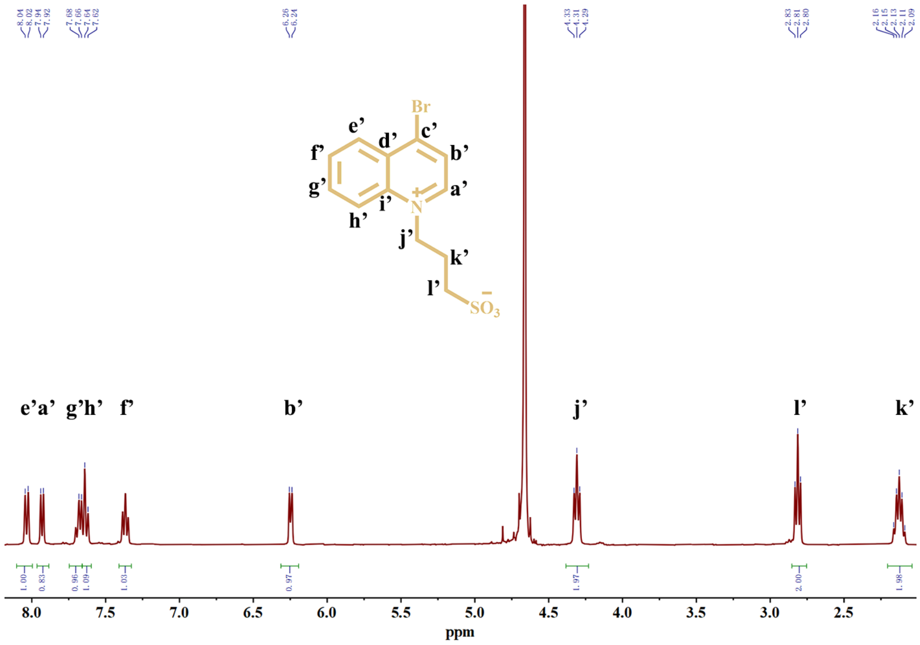

Figure S21. ^{1}H NMR (400 MHz, D_2O) spectrum of 4-Br-NaOH.

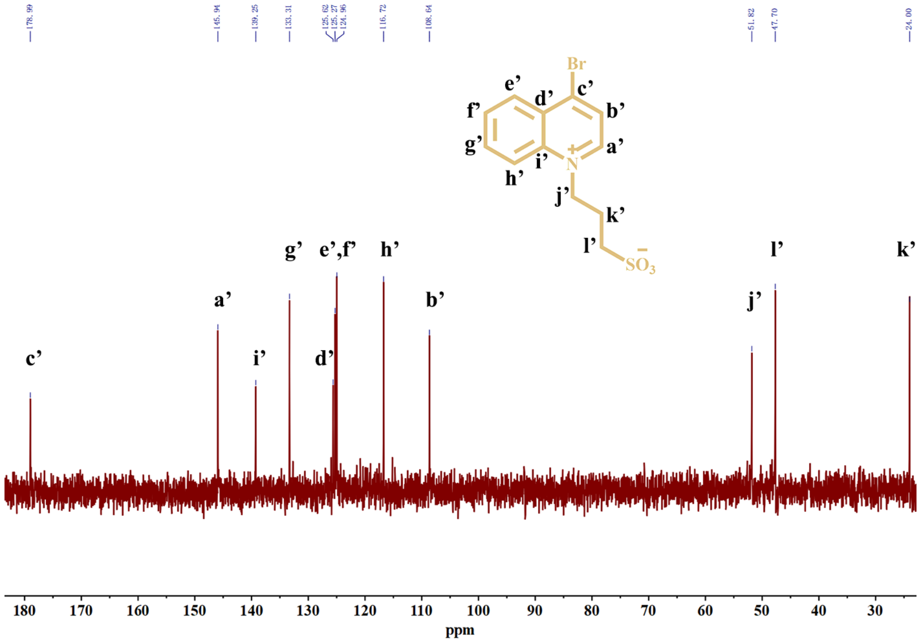

Figure S22. ^{13}C NMR (400 MHz, D_2O) spectrum of 4-Br-NaOH.

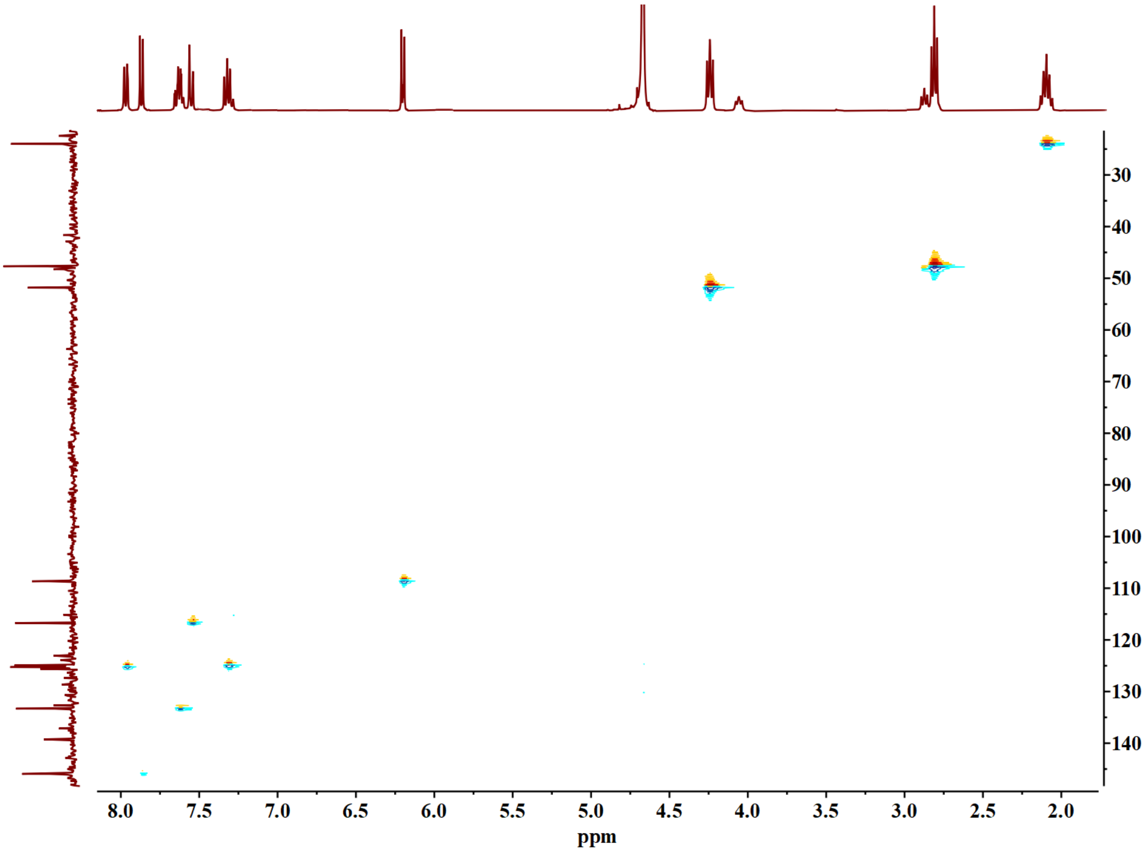

Figure S23. HSQC (400 MHz, D_2O) spectrum of 4-Br-NaOH.

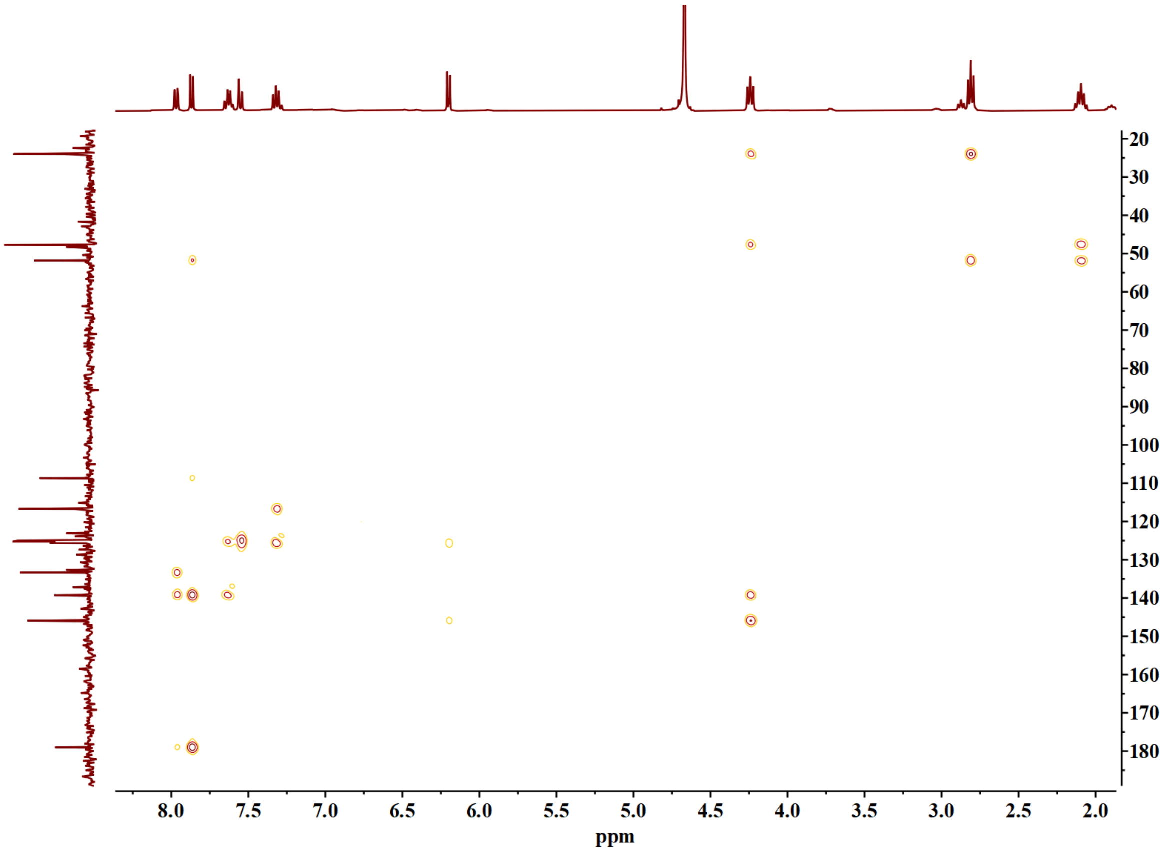

Figure S24. HMBC (400 MHz, D_2O) spectrum of 4-Br-NaOH.

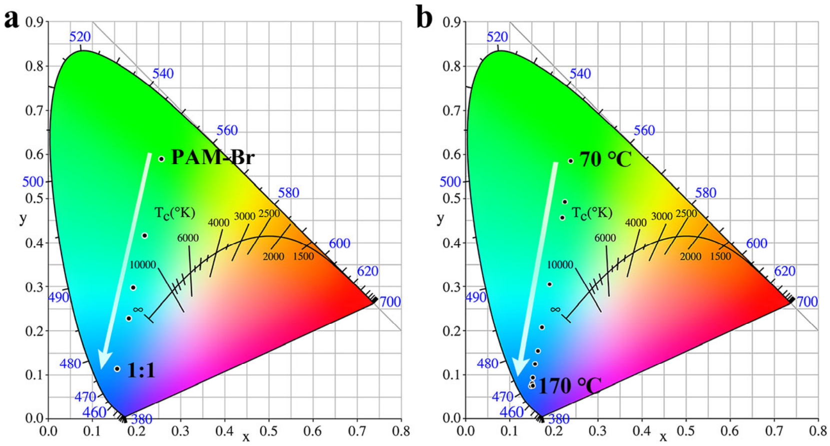

Figure S25. (a) Chromaticity coordinate (CIE) of PAM-Br with 0, 0.25, 0.5, 0.75, and 1 equivalent of NaOH. (b) CIE of PAM-Br after heating at different temperatures (70, 80, 90, 100, 110, 120, 130, 140, 150, 160 and 170 °C) for 10 min.

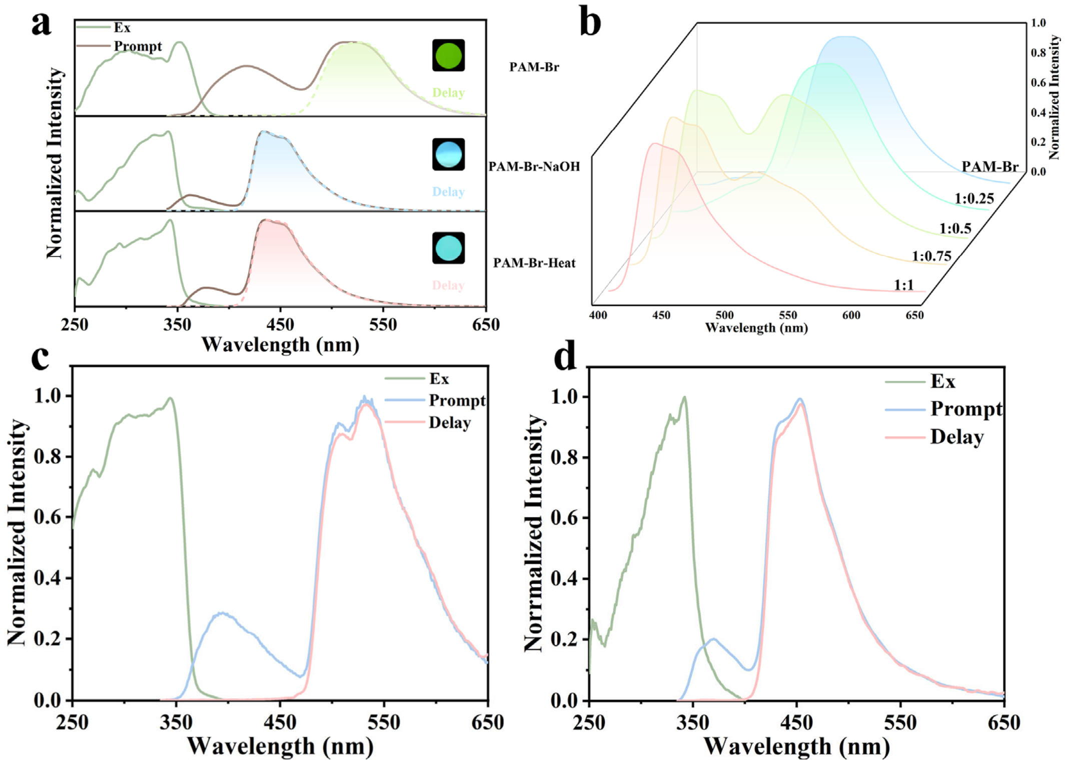

Figure S26. (a) Excitation spectra (green solid line), prompt (brown solid line) and delayed (dash line) phosphorescence spectra of PAM-Br, PAM-Br-NaOH and PAM-Br-Heat. Delayed time: 1.0 ms. (b) Normalized phosphorescence emission spectra of PAM-Br-NaOH with 0, 0.25, 0.50, 0.75, and 1 equivalent of NaOH. (c) Excitation spectra, prompt and delayed phosphorescence spectra of 4-Br (1×10^{-6} M) in methanol at 77 K. (d) Excitation spectra, prompt and delayed phosphorescence spectra of 4-Br-NaOH (1×10^{-6} M) in methanol at 77 K.

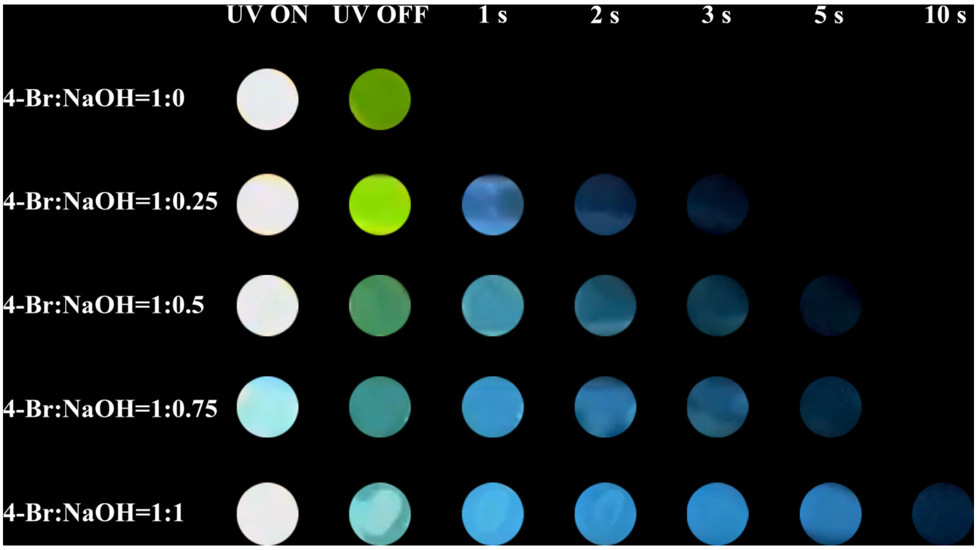

Figure S27. Afterglow images of PAM-Br-NaOH with different equivalents of NaOH (λ_{ex} = 365 nm) after ceasing irradiation in air environment.

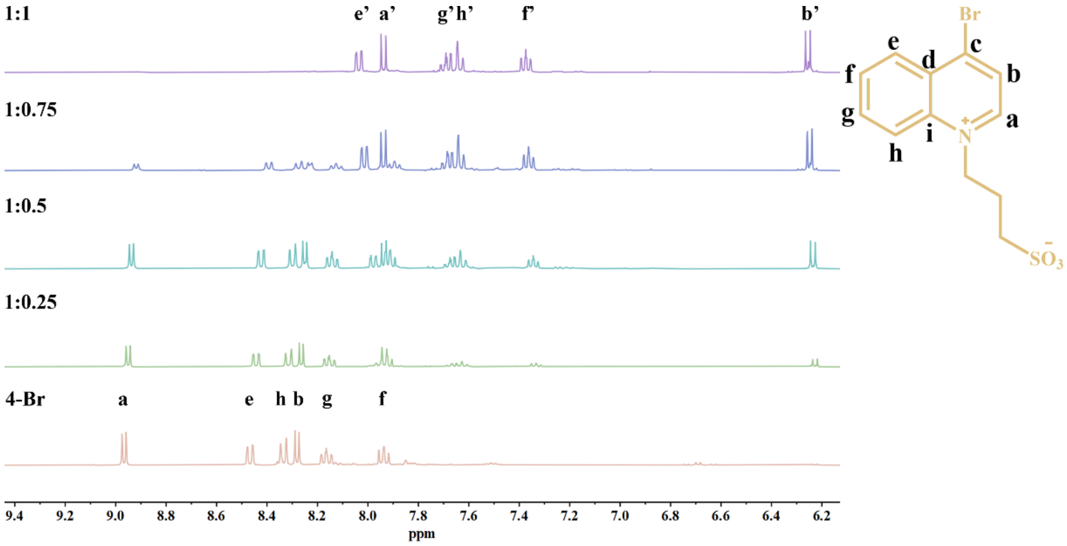

Figure S28. ^{1}H NMR spectra (400 MHz, D_2O) of 4-Br-NaOH with 0, 0.25, 0.5, 0.75, and 1 equivalent of NaOH.

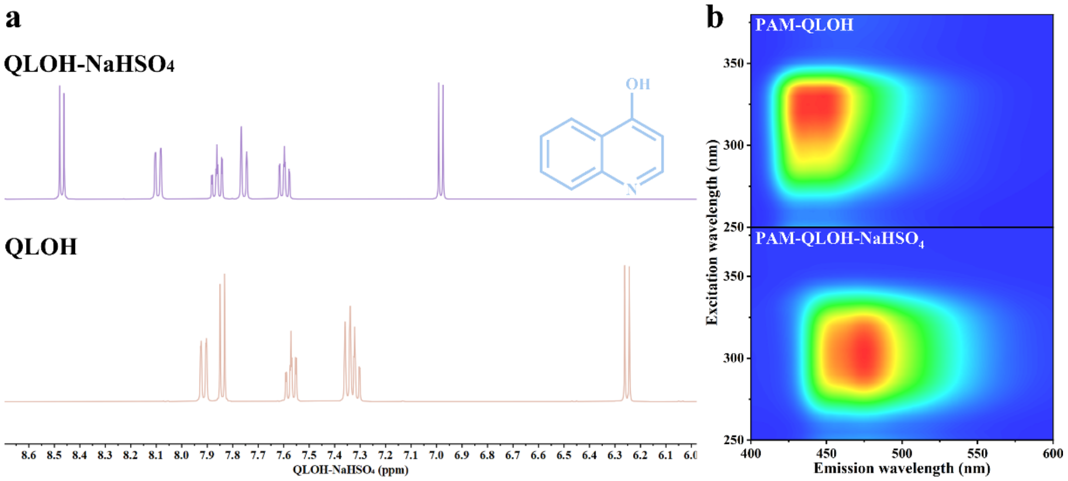

Figure S29. (a) ^{1}H NMR spectra (400 MHz, D_2O) of QLOH and QLOH with 1 equivalent of $NaHSO_4$. (b) Phosphorescence-excitation mapping of PAM-QLOH and PAM-QLOH-$NaHSO_4$.

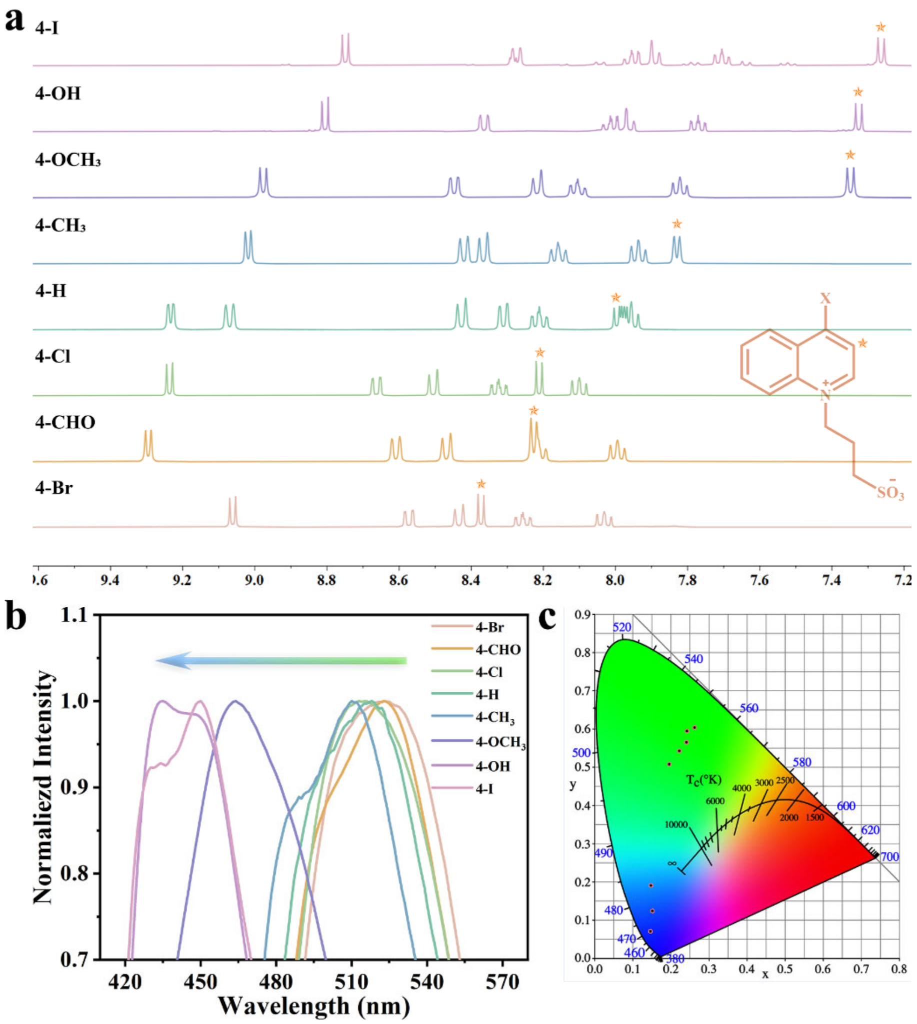

Figure S30. (a) ^{1}H NMR spectra (400 MHz, D_2O) of 4-X. (b) Normalized phosphorescence emission spectra of PAM-X. (c) CIE of PAM-X.

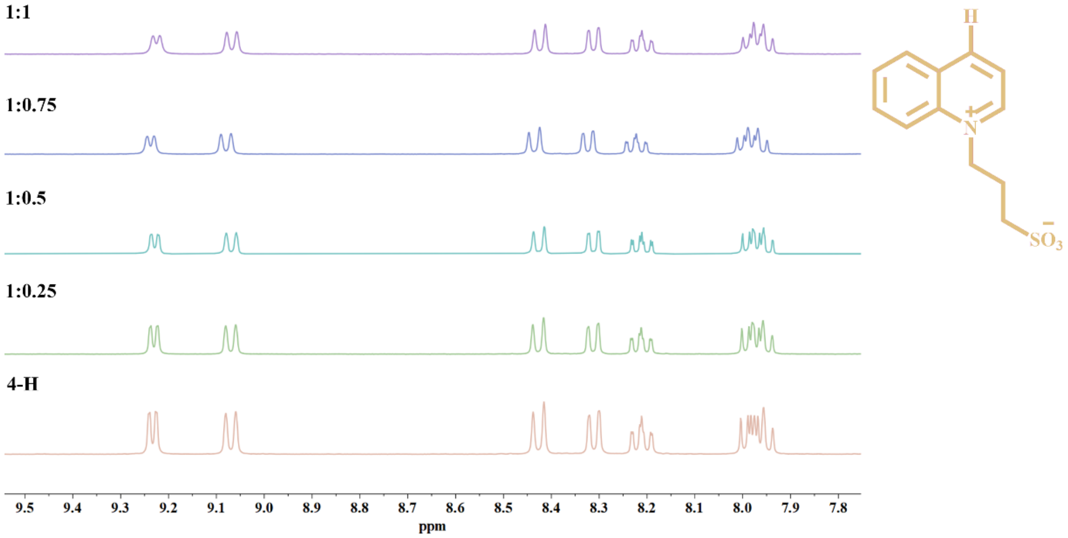

Figure S31. ^{1}H NMR spectra (400 MHz, D_2O) of 4-H-NaOH with 0, 0.25, 0.5, 0.75, and 1 equivalent of NaOH.

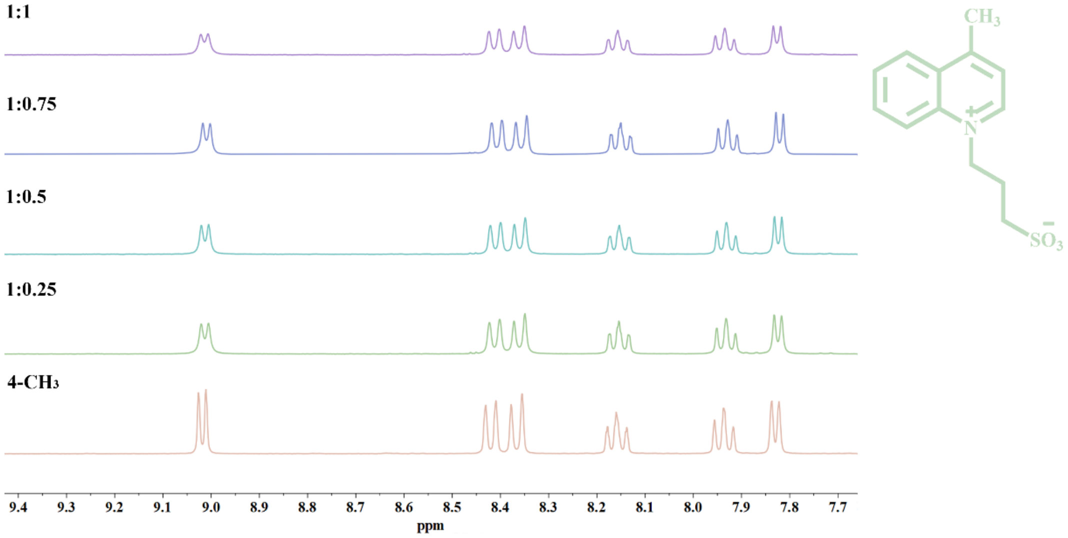

Figure S32. ^{1}H NMR spectra (400 MHz, D_2O) of 4-CH_3-NaOH with 0, 0.25, 0.5, 0.75, and 1 equivalent of NaOH.

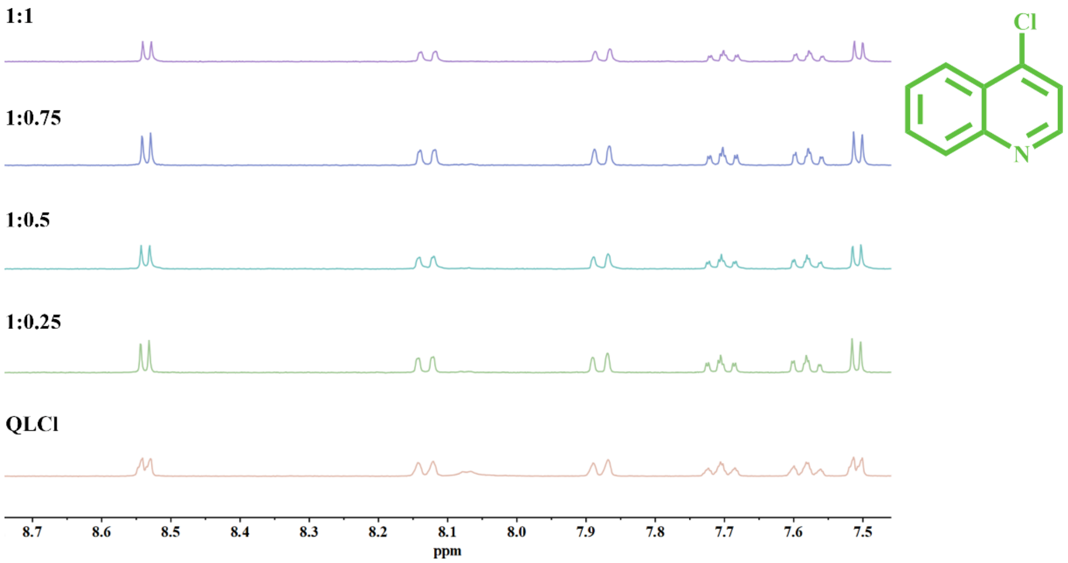

Figure S33. ^{1}H NMR spectra (400 MHz, D_2O) of QLCl-NaOH with 0, 0.25, 0.5, 0.75, and 1 equivalent of NaOH.

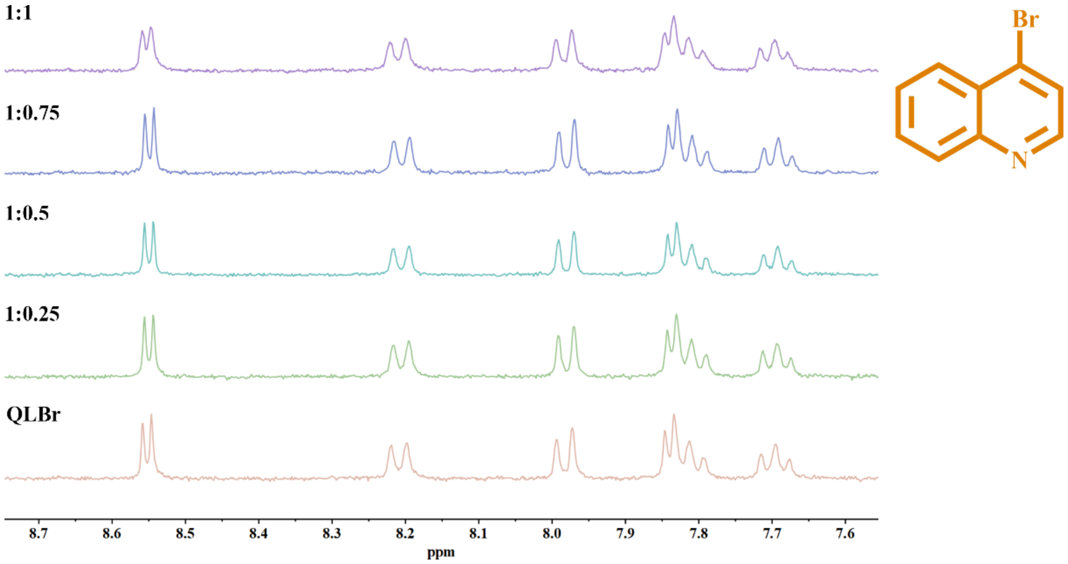

Figure S34. ^{1}H NMR spectra (400 MHz, D_2O) of QLBr-NaOH with 0, 0.25, 0.5, 0.75, and 1 equivalent of NaOH.

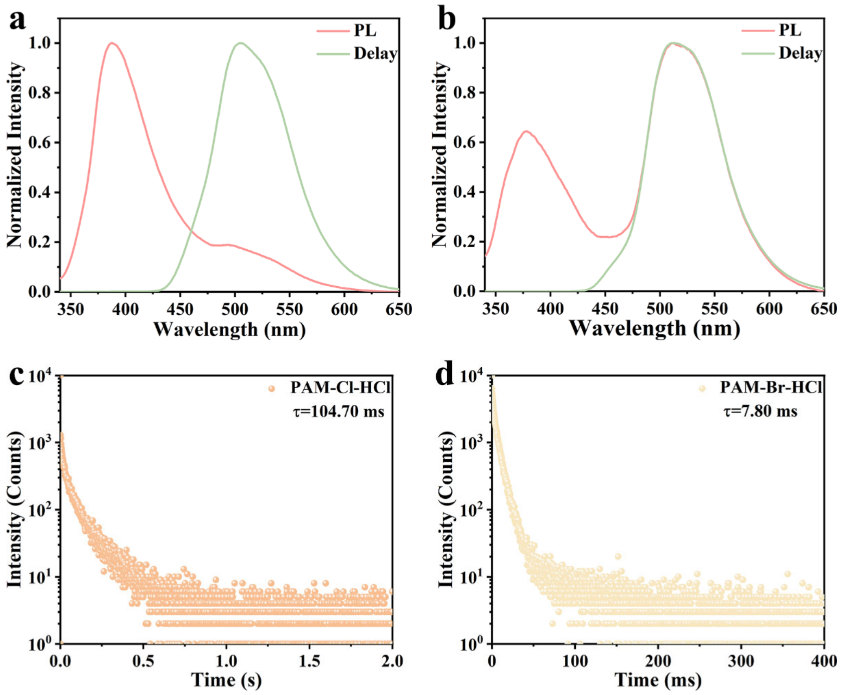

Figure S35. Normalized prompt (red line) and delayed (green line) phosphorescence spectra of PAM-Cl-HCl (a) and PAM-Br-HCl (b). Phosphorescence lifetime of PAM-Cl-HCl (c, excited at 350 nm, monitored at 520 nm) and PAM-Br-HCl (d, excited at 350 nm, monitored at 530 nm).

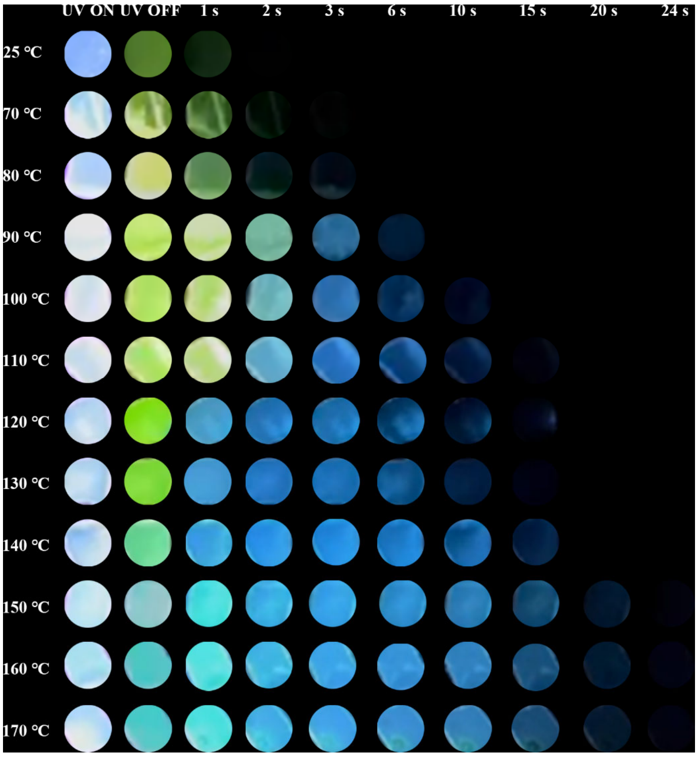

Figure S36. Afterglow images of PAM-Cl-Heat treated at different temperatures for 10 min (λ_{ex} = 365 nm) after ceasing irradiation in air environment.

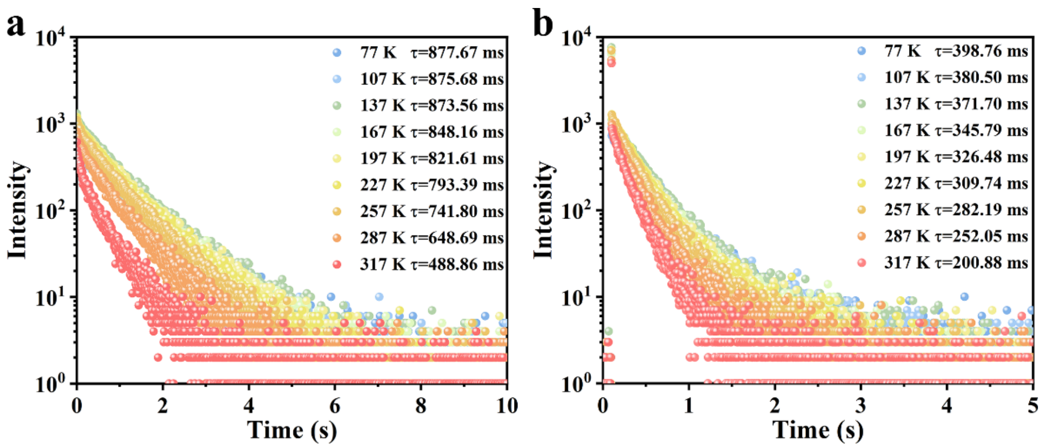

Figure S37. Temperature-dependent phosphorescence lifetime of PAM-Cl-Heat treated at 110 °C for 10 min from 77 to 317 K monitored at 434 nm (a) and 520 nm (b) excited at 340 nm.

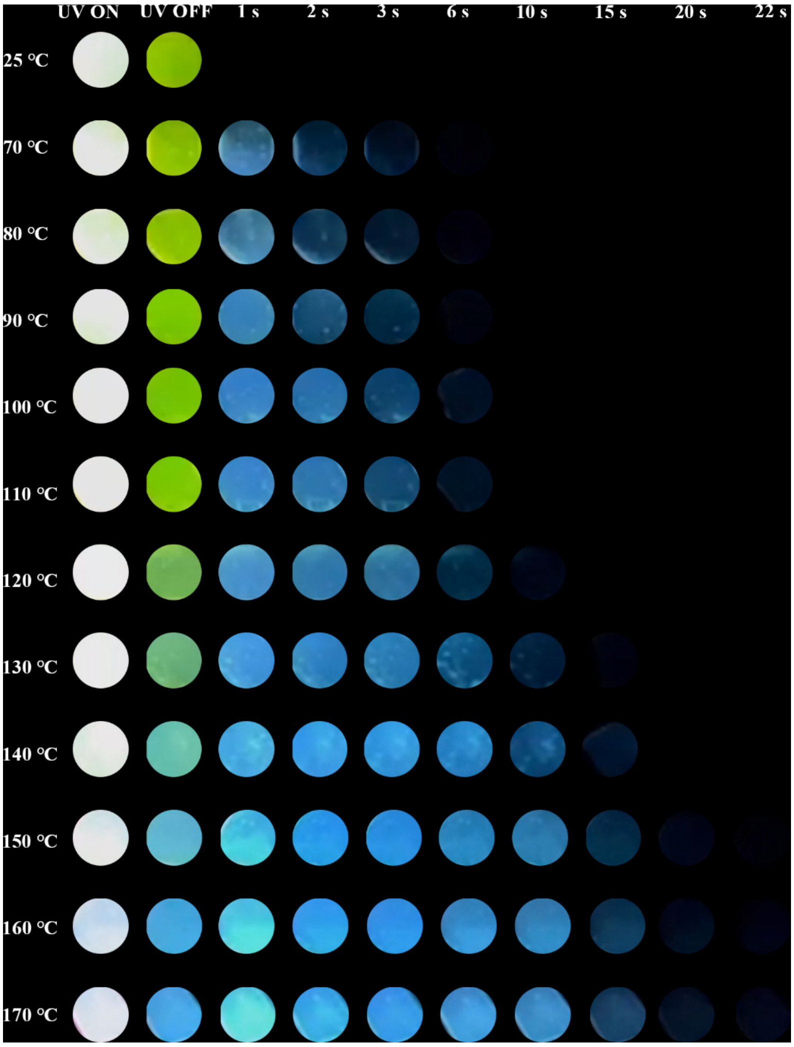

Figure S38. Afterglow images of PAM-Br-Heat treated at different temperatures for 10 min (λ_{ex} = 365 nm) after ceasing irradiation in air environment.

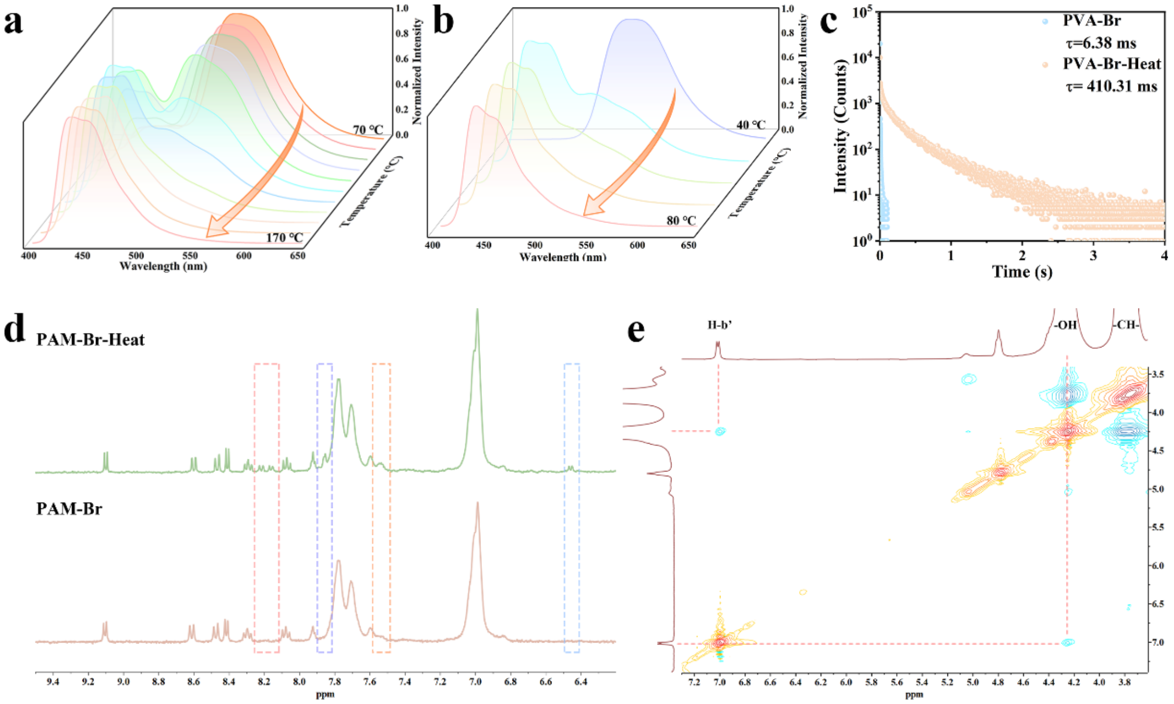

Figure S39. (a) Normalized phosphorescence emission spectra of PAM-Br-Heat treated at different temperatures for 10 min. (b) Normalized phosphorescence emission spectra of PVA-Br-Heat treated at different temperatures for 10 min. (c) Phosphorescence lifetime decay curves of PVA-Br (excited at 350 nm, monitored at 530 nm) and PVA-Br-Heat (excited at 340 nm, monitored at 434 nm). (d) ^{1}H NMR spectra (400 MHz, D_2O) of the mixture solution (down) of PAM and 4-Br and the solution after heating (up). (e) 2D NOESY NMR spectrum (400 MHz, d_6-DMSO) of PVA and 4-Br.

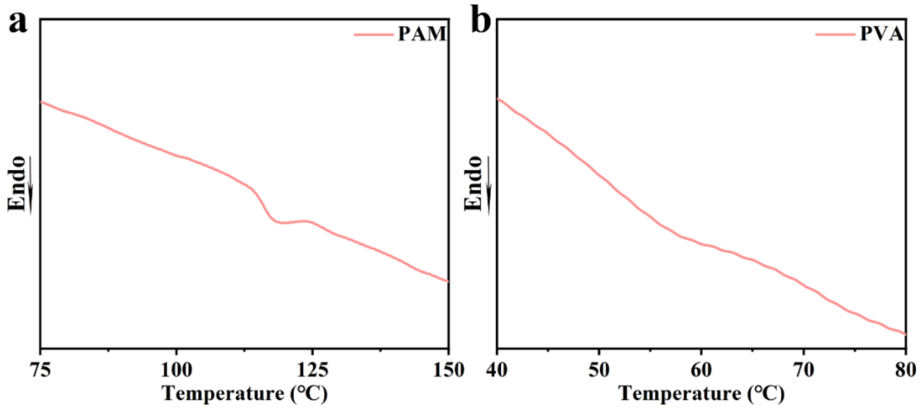

Figure S40. DSC curves of PAM (a) and PVA (b).

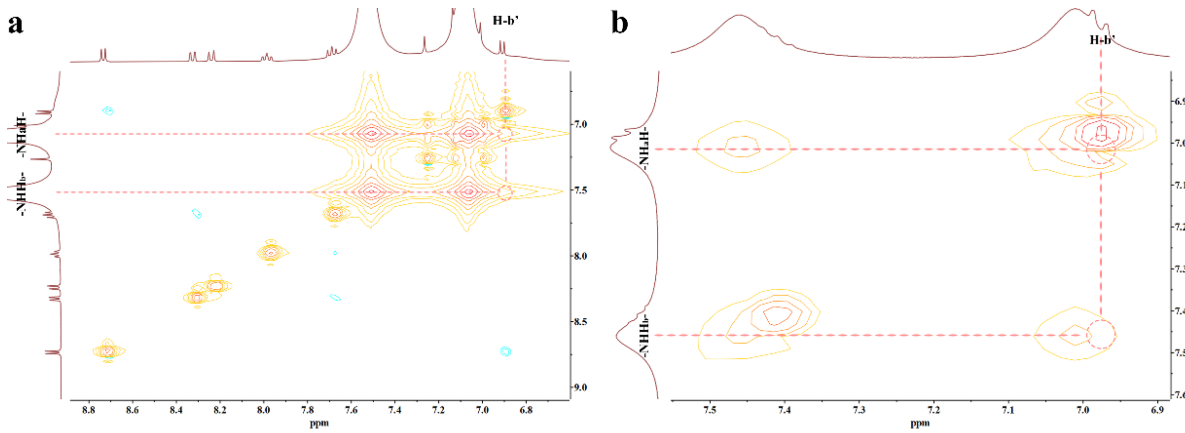

Figure S41. (a) 2D NOESY NMR spectrum (400 MHz, d_6-DMSO) of AM and 4-Cl after heating. (b) 2D NOESY NMR spectrum (400 MHz, d_6-DMSO) of AM and 4-Br after heating.

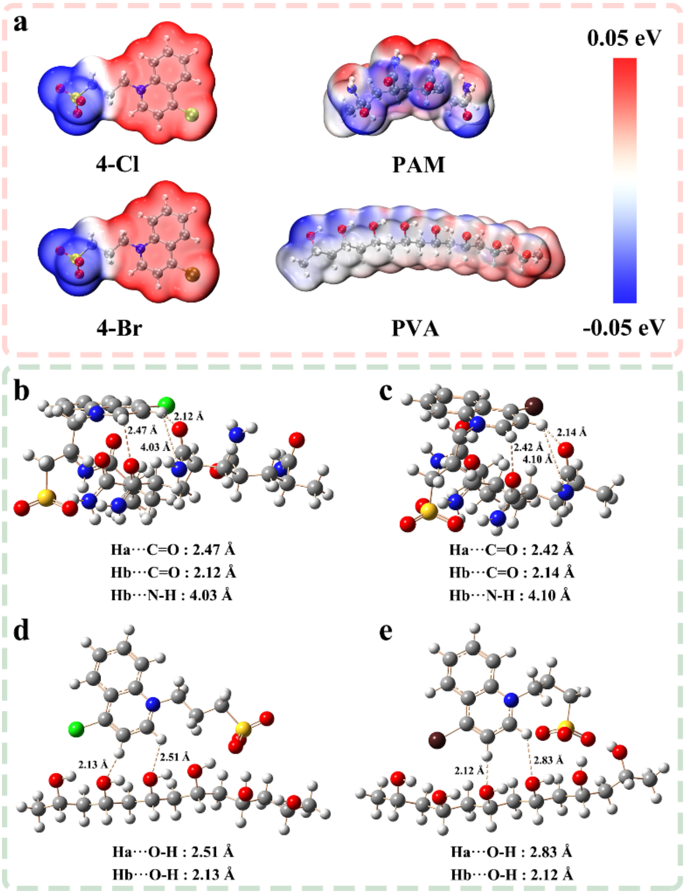

Figure S42. (a) Calculated ESP distribution of chromophores and polymers. The blue color implied areas with dense electron density, while the red areas indicated less electron density. Optimized molecular structures of PAM-Cl-Heat (b), PAM-Br-Heat (c), PVA-Cl-Heat (d) and PVA-Br-Heat (e).

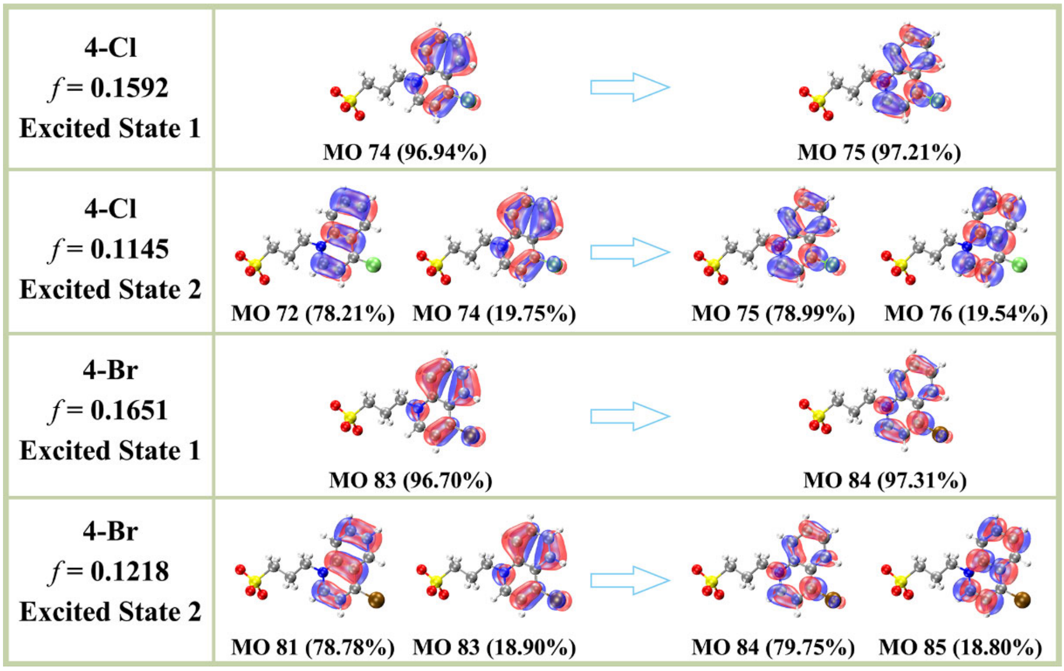

Figure S43. Electronic excitation analyzes of 4-Cl and 4-Br: visualization of dominant molecular orbitals, and their contribution to hole and electron distribution (%).

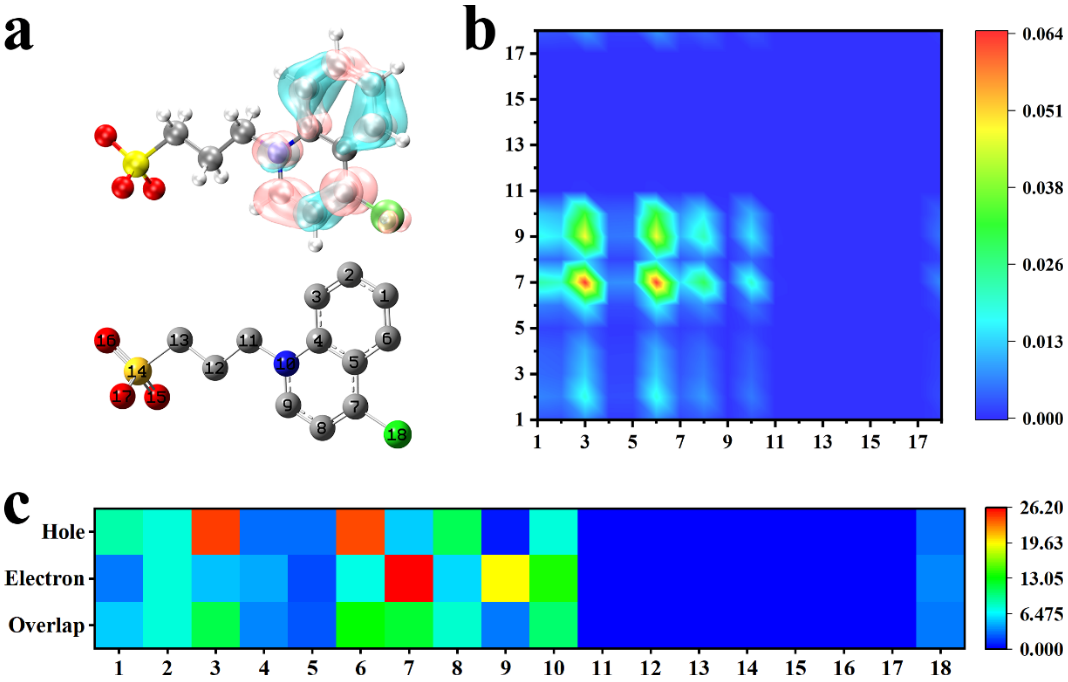

Figure S44. (a) Electron/hole map of the excited state 1 of 4-Cl (pink: electron; blue: hole) and the corresponding atomic number. (b) Thermal maps corresponding to the atom–atom charge transfer matrix of $S_0 \rightarrow S_1$ of 4-Cl. (c) The heat map of atoms' contribution to hole and electron.

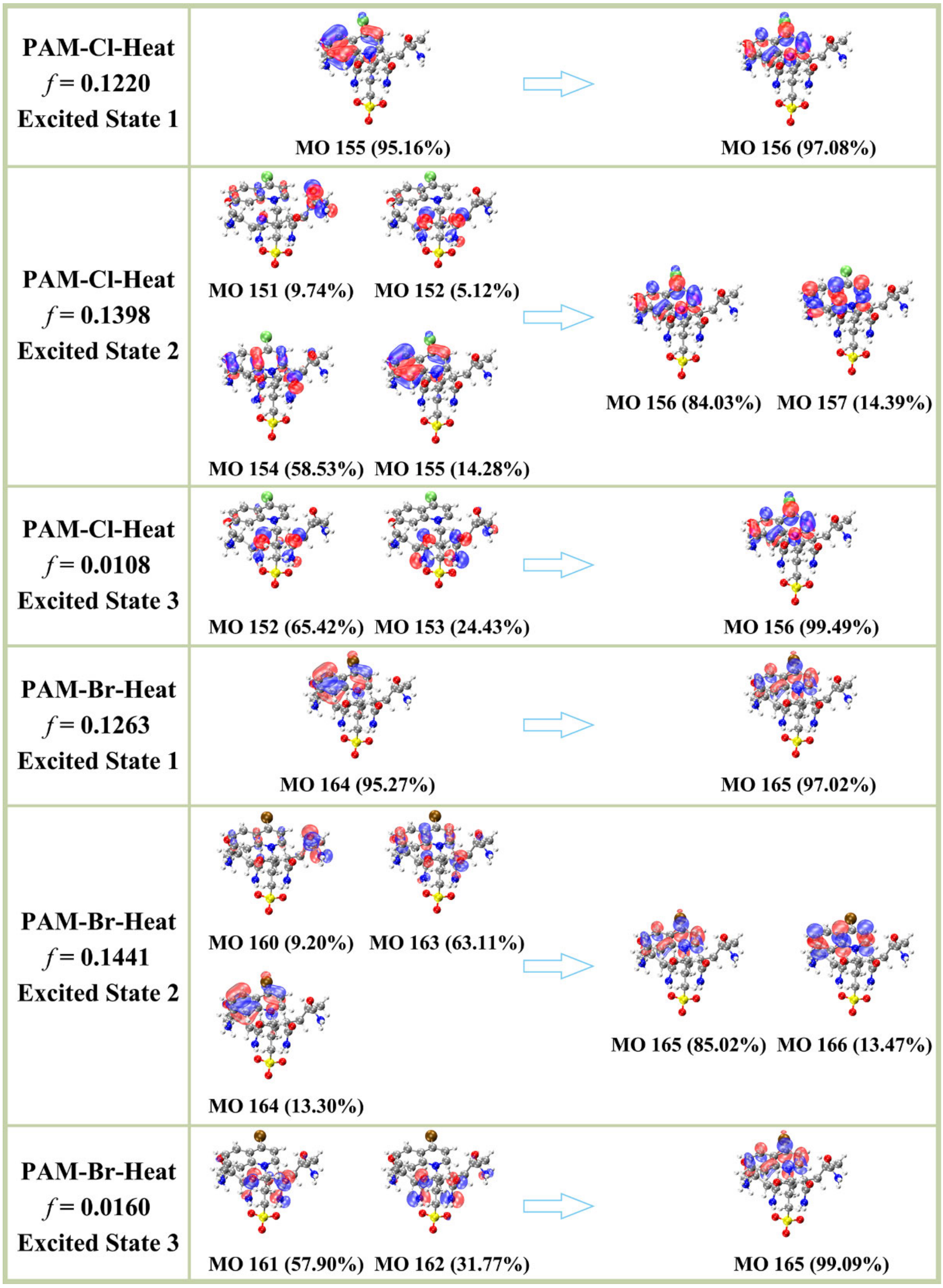

Figure S45. Electronic excitation analyzation of PAM-Cl-Heat and PAM-Br-Heat: visualization of dominant molecular orbitals, and their contribution to hole and electron distribution (%).

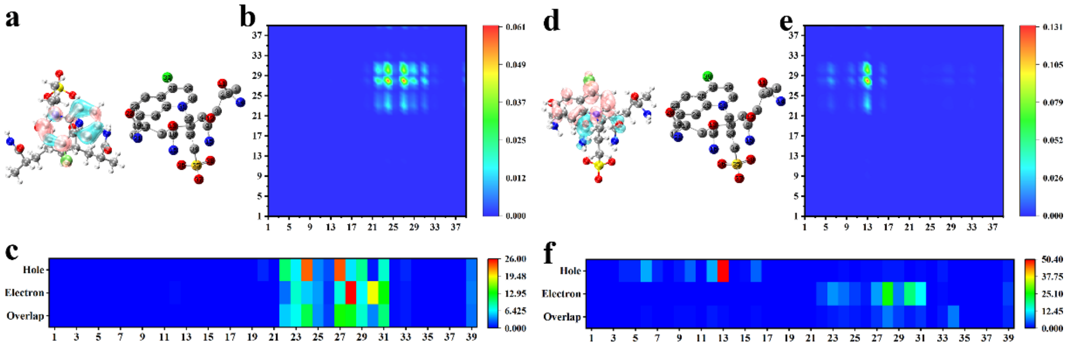

Figure S46. (a) Electron/hole map of the excited state 1 of PAM-Cl-Heat (pink: electron; blue: hole) and the corresponding atomic number. (b) Thermal maps corresponding to the atom–atom charge transfer matrix of $S_0 \rightarrow S_1$ for the excited state 1 of PAM-Cl-Heat. (c) The heat map of atoms' contribution to hole and electron for the excited state 1 of PAM-Cl-Heat. (d) Electron/hole map of the excited state 3 of PAM-Cl-Heat (pink: electron; blue: hole) and the corresponding atomic number. (e) Thermal maps corresponding to the atom–atom charge transfer matrix of $S_0 \rightarrow S_1$ for the excited state 3 of PAM-Cl-Heat. (f) The heat map of atoms' contribution to hole and electron for the excited state 3 of PAM-Cl-Heat.

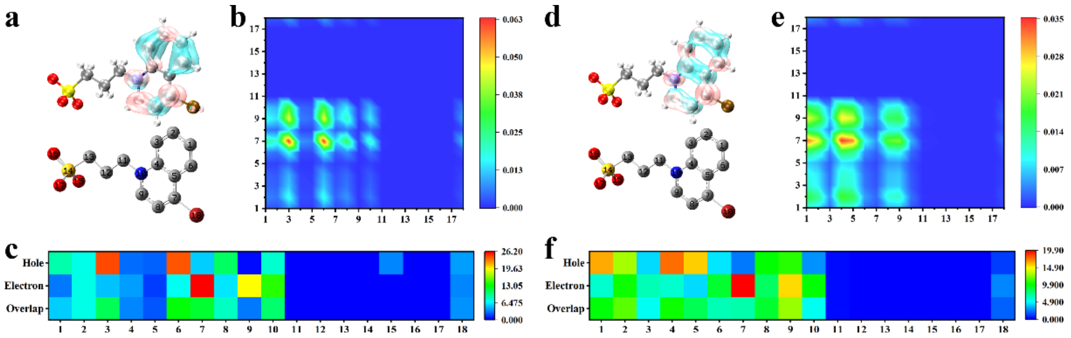

Figure S47. (a) Electro/hole map of the excited state 1 of 4-Br (pink: electron; blue: hole) and the corresponding atomic number. (b) Thermal maps corresponding to the atom–atom charge transfer matrix of $S_0 \rightarrow S_1$ for the excited state 1 of 4-Br. (c) The heat map of atoms' contribution to hole and electron for the excited state 1 of 4-Br. (d) Electro/hole map of the excited state 2 of 4-Br (pink: electron; blue: hole) and the corresponding atomic number. (e) Thermal maps corresponding to the atom–atom charge transfer matrix of $S_0 \rightarrow S_1$ for the excited state 2 of 4-Br. (f) The heat map of atoms' contribution to hole and electron for the excited state 2 of 4-Br.

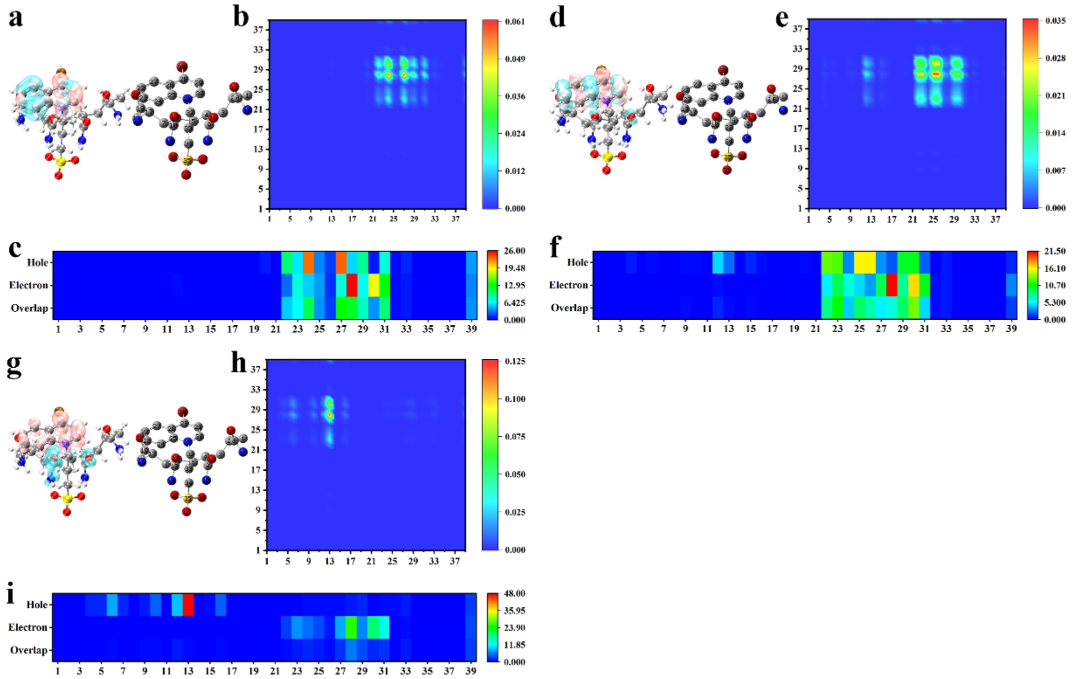

Figure S48. (a) Electron/hole map of the excited state 1 of PAM-Br-Heat (pink: electron; blue: hole) and the corresponding atomic number. (b) Thermal maps corresponding to the atom–atom charge transfer matrix of $S_0 \rightarrow S_1$ for the excited state 1 of PAM-Br-Heat. (c) The heat map of atoms' contribution to hole and electron for the excited state 1 of PAM-Br-Heat. (d) Electron/hole map of the excited state 2 of PAM-Br-Heat (pink: electron; blue: hole) and the corresponding atomic number. (e) Thermal maps corresponding to the atom–atom charge transfer matrix of $S_0 \rightarrow S_1$ for the excited state 2 of PAM-Br-Heat. (f) The heat map of atoms' contribution to hole and electron for the excited state 2 of PAM-Br-Heat. (g) Electron/hole map of the excited state 3 of PAM-Br-Heat (pink: electron; blue: hole) and the corresponding atomic number. (h) Thermal maps corresponding to the atom–atom charge transfer matrix of $S_0 \rightarrow S_1$ for the excited state 3 of PAM-Br-Heat. (i) The heat map of atoms' contribution to hole and electron for the excited state 3 of PAM-Br-Heat.

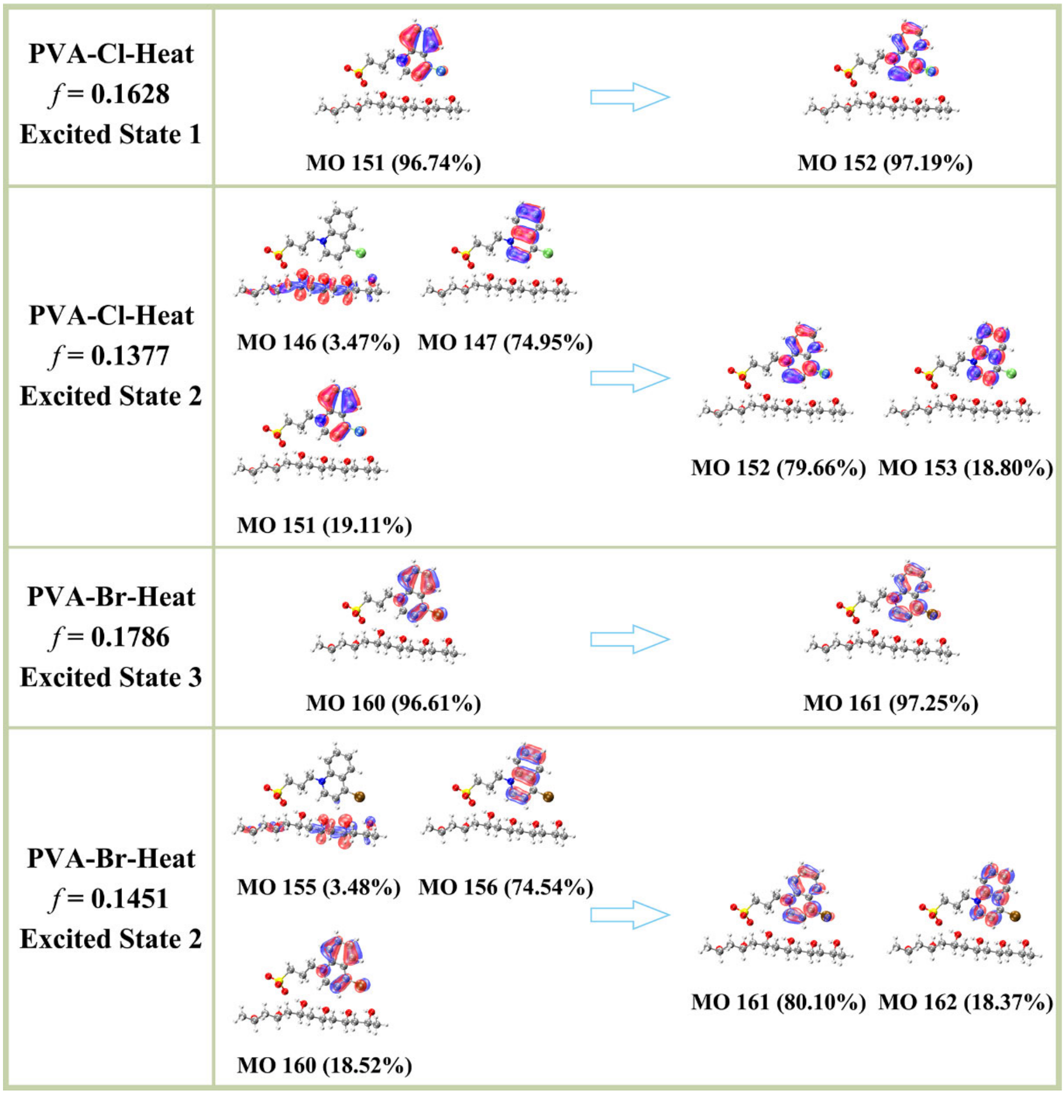

Figure S49. Electronic excitation analyzes of PVA-Cl-Heat and PVA-Br-Heat: visualization of dominant molecular orbitals, and their contribution to hole and electron distribution (%).

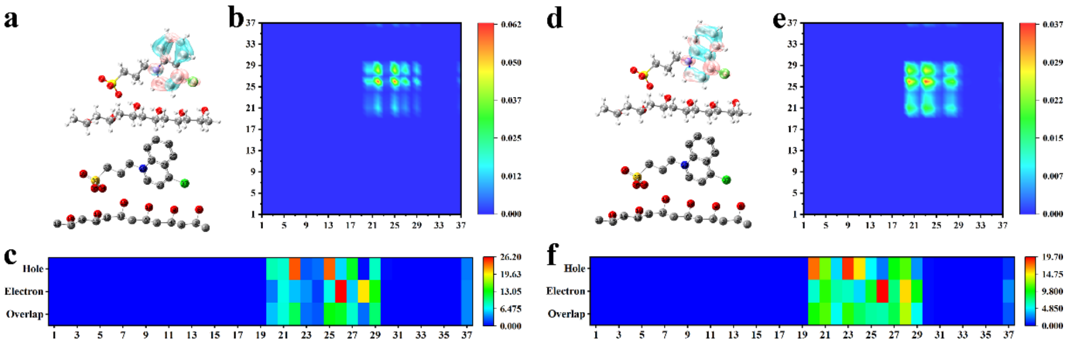

Figure S50. (a) Electro/hole map of the excited state 1 of PVA-Cl-Heat (pink: electron; blue: hole) and the corresponding atomic number. (b) Thermal maps corresponding to the atom–atom charge transfer matrix of $S_0 \rightarrow S_1$ for the excited state 1 of PVA-Cl-Heat. (c) The heat map of atoms' contribution to hole and electron for the excited state 1 of PVA-Cl-Heat. (d) Electron/hole map of the excited state 2 of PVA-Cl-Heat (pink: electron; blue: hole) and the corresponding atomic number. (e) Thermal maps corresponding to the atom–atom charge transfer matrix of $S_0 \rightarrow S_1$ for the excited state 2 of PVA-Cl-Heat. (f) The heat map of atoms' contribution to hole and electron for the excited state 2 of PVA-Cl-Heat.

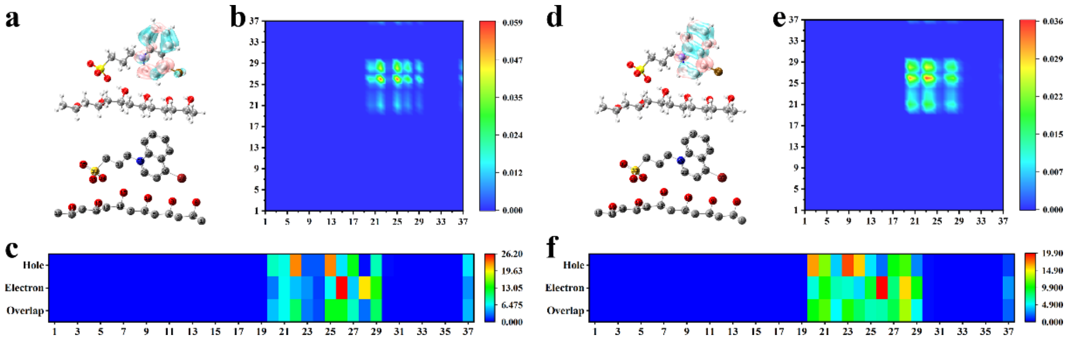

Figure S51. (a) Electron/hole map of the excited state 1 of PVA-Br-Heat (pink: electron; blue: hole) and the corresponding atomic number. (b) Thermal maps corresponding to the atom–atom charge transfer matrix of $S_0 \rightarrow S_1$ for the excited state 1 of PVA-Br-Heat. (c) The heat map of atoms' contribution to hole and electron for the excited state 1 of PVA-Br-Heat. (d) Electron/hole map of the excited state 2 of PVA-Br-Heat (pink: electron; blue: hole) and the corresponding atomic number. (e) Thermal maps corresponding to the atom–atom charge transfer matrix of $S_0 \rightarrow S_1$ for the excited state 2 of PVA-Br-Heat. (f) The heat map of atoms' contribution to hole and electron for the excited state 2 of PVA-Br-Heat.

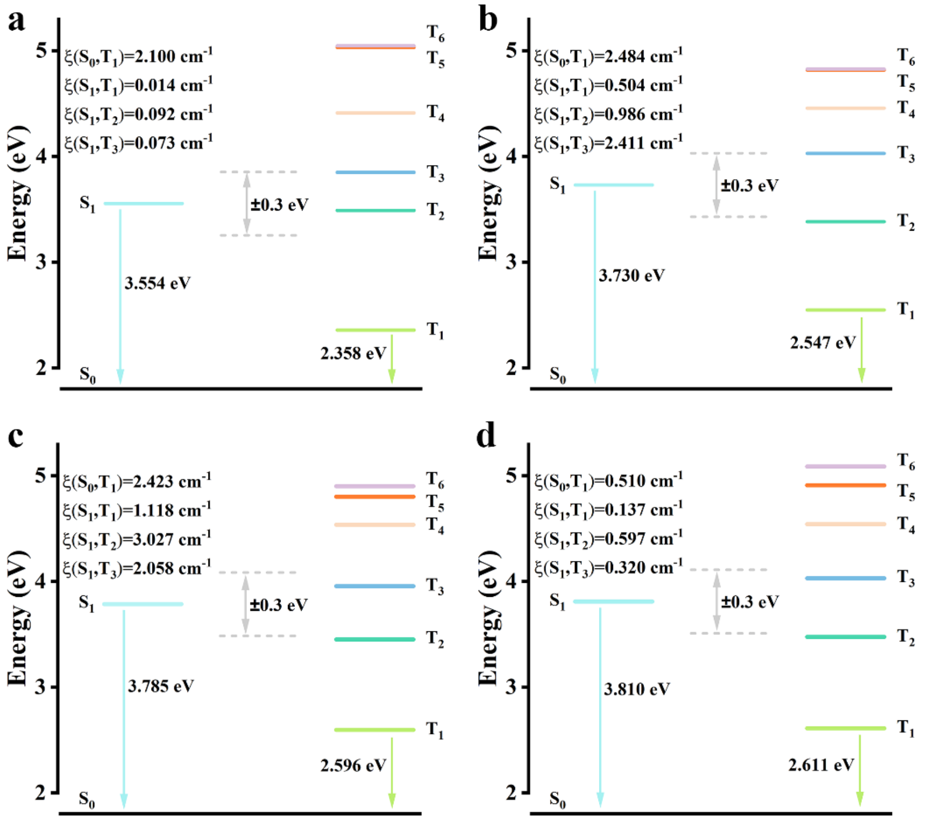

Figure S52. Diagrams of the TD-DFT calculated energy levels and SOC constants of 4-Br (a), PAM-Br-Heat (b), PVA-Br-Heat (c) and PVA-Cl-Heat (d).

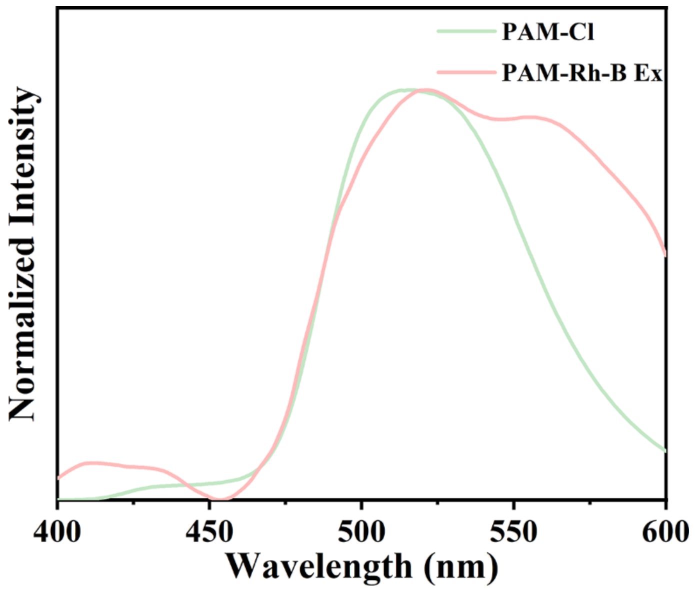

Figure S53. Excitation spectrum of PAM-Rh-B and delayed PL spectrum of PAM-Cl.

Table S1. Photophysical properties of PAM-Cl, PAM-Cl-NaOH, PAM-Cl-Heat, PAM-Br, PAM-Br-NaOH and PAM-Br-Heat.

Sample	τ_F (ns)	Φ_F (%)	τ_P (ms)	Φ_P (%)	k_r^F (s^{-1})	Φ_{ISC} (%)	k_{ISC} (s^{-1})	k_r^P (s^{-1})	k_{nr}^P (s^{-1})
PAM-Cl	6.78	11.10	171.8	1.76	1.64×10^7	88.90	2.60×10^6	0.10	5.72
PAM-Cl-NaOH	0.77	2.82	602.7	28.64	3.66×10^7	97.18	3.72×10^8	0.48	1.18
PAM-Cl-Heat	5.59	2.08	629.9	20.51	3.72×10^6	97.92	3.67×10^7	0.33	1.26
PAM-Br	4.54	1.95	12.3	3.31	4.30×10^6	98.05	7.29×10^6	2.69	78.61
PAM-Br-NaOH	1.03	2.66	589.3	19.9	2.58×10^7	97.40	1.93×10^8	0.34	1.36
PAM-Br-Heat	3.15	1.73	613.6	12.21	5.49×10^6	98.27	3.88×10^7	0.20	1.43

$k_r^P + k_{nr}^P = 1/\tau_P$ (S1)

$k_r^F = \Phi_F/\tau_F$ (S2)

$\Phi_{ISC} = 1-\Phi_F-\Phi_{IC} \approx 1-\Phi_F$ (S3)

$k_{ISC} = \Phi_P/\tau_F$ (S4)

$k_r^P = \Phi_P/\tau_P$ (S5)

$k_{nr}^P = (1-\Phi_P)/\tau_P$ (S6)

References:

[1] Frisch, M. J.; Trucks, G. W.; Schlegel, H. B.; Scuseria, G. E.; Robb, M. A.; Cheeseman, J. R.; Scalmani, G.; Barone, V.; Mennucci, B.; Petersson, G. A.; Nakatsuji, H.; Caricato, M.; Li, X.; Hratchian, H. P.; Izmaylov, A. F.; Bloino, J.; Zheng, G.; Sonnenberg, J. L.; Hada, M.; Ehara, M.; Toyota, K.; Fukuda, R.; Hasegawa, J.; Ishida, M.; Nakajima, T.; Honda, Y.; Kitao, O.; Nakai, H.; Vreven, T.; Montgomery Jr, J. A.; Peralta, J. E.; Ogliaro, F.; Bearpark, M. J.; Heyd, J.; Brothers, E. N.; Kudin, K. N.; Staroverov, V. N.; Kobayashi, R.; Normand, J.; Raghavachari, K.; Rendell, A. P.; Burant, J. C.; Iyengar, S. S.; Tomasi, J.; Cossi, M.; Rega, N.; Millam, N. J.; Klene, M.; Knox, J. E.; Cross, J. B.; Bakken, V.; Adamo, C.; Jaramillo, J.; Gomperts, R.; Stratmann, R. E.; Yazyev, O.; Austin, A. J.; Cammi, R.; Pomelli, C.; Ochterski, J. W.; Martin, R. L.; Morokuma, K.; Zakrzewski, V. G.; Voth, G. A.; Salvador, P.; Dannenberg, J. J.; Dapprich, S.; Daniels,

A. D.; Farkas, Ö.; Foresman, J. B.; Ortiz, J. V.; Cioslowski, J.; Fox, D. J., Gaussian 09, Revision E.01; Gaussian, Inc., Wallingford CT, 2013.

[2] Y. Zhao, D. G. Truhlar, *Theor. Chem. Acc.* **2008**, *120*, 215-241.

[3] F. Weigend, R. Ahlrichs, *Phys. Chem. Chem. Phys.* **2005**, *7*, 3297-3305.

[4] T. Lu, F. Chen, *J. Comput. Chem.* **2012**, *33*, 580-592.

[5] W. Humphrey, A. Dalke, K. Schulten, *J. Mol. Graph.* **1996**, *14*, 33-38.

[6] F. Neese, *WIREs Comput. Mol. Sci.* **2012**, *2*, 73-78.

[7] F. Weigend, *Phys. Chem. Chem. Phys.* **2006**, *8*, 1057-1065.

[8] (a) E. Haimov, A. Chapman, F. Bresme, A. S. Holmes, T. Reddyhoff, M. Urbakh, A. A. Kornyshev, *Nat. Commun.* **2023**, *14*, 483; (b) H. Peng, G. Xie, Y. Cao, L. Zhang, X. Yan, X. Zhang, S. Miao, Y. Tao, H. Li, C. Zheng, W. Huang, R. Chen, *Sci. Adv.* **2022**, *8*, eabk2925.
